# Identification of novel protein biomarkers and drug targets for colorectal cancer by integrating human plasma proteome with genome

**DOI:** 10.1186/s13073-023-01229-9

**Published:** 2023-09-19

**Authors:** Jing Sun, Jianhui Zhao, Fangyuan Jiang, Lijuan Wang, Qian Xiao, Fengyan Han, Jie Chen, Shuai Yuan, Jingsun Wei, Susanna C. Larsson, Honghe Zhang, Malcolm G Dunlop, Susan M Farrington, Kefeng Ding, Evropi Theodoratou, Xue Li

**Affiliations:** 1 Department of Big Data in Health Science School of Public Health, and Center of Clinical Big Data and Analytics of The Second Affiliated Hospital, Zhejiang University School of Medicine, Hangzhou, Zhejiang China; 2 Centre for Global Health, Usher Institute, University of Edinburgh, Edinburgh, UK; 3 Colorectal Surgery and Oncology, Key Laboratory of Cancer Prevention and Intervention, Ministry of Education, The Second Affiliated Hospital, Zhejiang University School of Medicine, Hangzhou, China; 4 Department of Pathology and Women’s Hospital, Zhejiang University School of Medicine, Hangzhou, Zhejiang China; 5 Unit of Cardiovascular and Nutritional Epidemiology, Institute of Environmental Medicine, Karolinska Institutet, Stockholm, Sweden; 6 Unit of Medical Epidemiology, Department of Surgical Sciences, Uppsala University, Uppsala, Sweden; 7 Cancer Research UK Edinburgh Centre, Medical Research Council Institute of Genetics and Cancer, University of Edinburgh, Edinburgh, UK; 8 Colon Cancer Genetics Group, Institute of Genetics and Cancer, University of Edinburgh, Edinburgh, UK

**Keywords:** Colorectal cancer, Protein, Proteome-wide Mendelian randomization, Biomarker, Drug target

## Abstract

**Background:**

The proteome is a major source of therapeutic targets. We conducted a proteome-wide Mendelian randomization (MR) study to identify candidate protein markers and therapeutic targets for colorectal cancer (CRC).

**Methods:**

Protein quantitative trait loci (pQTLs) were derived from seven published genome-wide association studies (GWASs) on plasma proteome, and summary-level data were extracted for 4853 circulating protein markers. Genetic associations with CRC were obtained from a large-scale GWAS meta-analysis (16,871 cases and 26,328 controls), the FinnGen cohort (4957 cases and 304,197 controls), and the UK Biobank (9276 cases and 477,069 controls). Colocalization and summary-data-based MR (SMR) analyses were performed sequentially to verify the causal role of candidate proteins. Single cell-type expression analysis, protein-protein interaction (PPI), and druggability evaluation were further conducted to detect the specific cell type with enrichment expression and prioritize potential therapeutic targets.

**Results:**

Collectively, genetically predicted levels of 13 proteins were associated with CRC risk. Elevated levels of two proteins (GREM1, CHRDL2) and decreased levels of 11 proteins were associated with an increased risk of CRC, among which four (GREM1, CLSTN3, CSF2RA, CD86) were prioritized with the most convincing evidence. These protein-coding genes are mainly expressed in tissue stem cells, epithelial cells, and monocytes in colon tumor tissue. Two interactive pairs of proteins (GREM1 and CHRDL2; MMP2 and TIMP2) were identified to be involved in osteoclast differentiation and tumorigenesis pathways; four proteins (POLR2F, CSF2RA, CD86, MMP2) have been targeted for drug development on autoimmune diseases and other cancers, with the potentials of being repurposed as therapeutic targets for CRC.

**Conclusions:**

This study identified several protein biomarkers to be associated with CRC risk and provided new insights into the etiology and promising targets for the development of screening biomarkers and therapeutic drugs for CRC.

**Supplementary Information:**

The online version contains supplementary material available at 10.1186/s13073-023-01229-9.

## Background

Colorectal cancer (CRC) is the third most common malignancy and the second leading cause of cancer death, with 1.9 million new cases and 0.9 million deaths worldwide in 2020 [1]. The CRC survival remains to be improved via early detection or targeted anticancer therapy [2]. Further evidence regarding non-invasive early diagnostic biomarkers and the development of novel therapeutic targets for CRC is urgently required.

Proteins, appearing in blood circulation due to cellular leakage or active secretion, provide a window into the human health state [3] and act as a major source of biomarkers and druggable targets [4]. Previous studies have found several circulating proteins to be associated with CRC risk [5–9]. However, most of these studies were limited as candidate approach with a few numbers of proteins, observational design, or small sample size, which limited opportunities to understand the causal role of protein makers in CRC risk.

Large-scale proteomic studies have identified over 18,000 protein quantitative trait loci (pQTLs) covering more than 4800 proteins, including over 1800 independent cis pQTLs [10–16]. These studies provide valuable data resources to systematically elucidate the causal effects of plasma proteins on CRC risk by Mendelian randomization (MR). MR uses genetic variants that are naturally randomized at conception as a natural experiment to uncover causal relationships of exposures with diseases, minimizing the chance of reverse causation and confounding bias [17]. Proteome-wide MR has recently offered important insights into understanding the etiology and prioritizing druggable targets for stroke, diabetes, psychiatric disorders, and ovarian cancer [18–22].

In this study, we performed a proteome-wide MR analysis by integrating human plasma proteome with genome data to systematically identify circulating protein biomarkers associated with CRC risk. Considering that MR alone may be insufficient in identifying credible proteins on causal pathways to cancer, colocalization, summary-data-based MR (SMR), and the HEIDI test were subsequently performed. Single cell-type expression analysis was employed to detect their enrichment cell type in colon tumor tissue. Lastly, druggability evaluation was performed to explore their potential as therapeutic targets for CRC.

## Methods

**Fig. 1:**
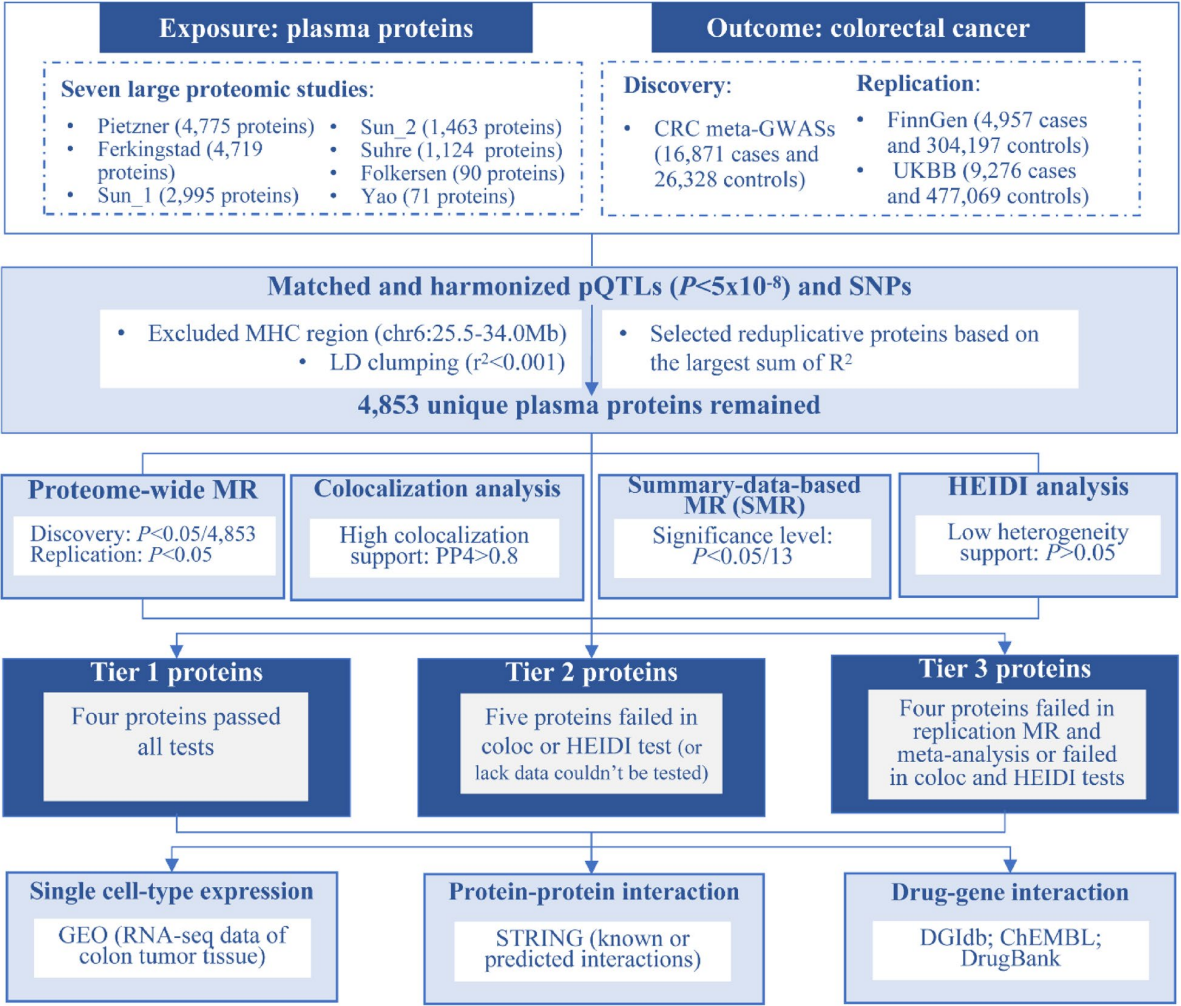
Flowchart of the study design

The overall study design is shown in Fig. 1. Briefly, we employed pQTL data derived from seven large-scale proteomic studies and examined their associations with CRC using a two-stage (discovery and replication) proteome-wide MR framework. Bayesian colocalization, summary-data-based MR (SMR), and HEIDI tests were leveraged to verify the causal associations between protein biomarkers and CRC. Single cell-type expression analysis was further conducted to detect the specific cell type of colon tumor tissue in which targeted protein-coding genes had enrichment expression. Last, protein-protein interaction (PPI) and druggability evaluation of identified protein biomarkers were performed to prioritize the potential therapeutic targets.

### Study population and datasets

The current study included CRC cases and controls of European ancestry from a meta-analysis of 11 previously published CRC GWASs [23]. Details for the study population, genotyping, and imputation information have been described elsewhere [23]. After standard quality control (QC), a total of 16,871 CRC cases and 26,328 controls were included in the discovery proteome-wide Mendelian randomization (MR) analysis. Two GWAS summary data included CRC cases and controls of European ancestry from independent FinnGen (4957 CRC cases and 304,197 controls) [24] and UK Biobank (UKBB) (9276 cases and 477,069 controls) [25] datasets were employed in the replication proteome-wide MR. In stratified analysis by tumor site (colon or rectum), 3793 colon cancer cases and 410,350 controls of European ancestry; 2091 rectal cancer cases and 410,350 controls of European ancestry were included [26]. All participants provided informed consent, and the ethics approvals were obtained from the relevant authorities. The basic information of these datasets is shown in Additional file 1: Table S1.

### Proteomic data source

Seven large-scaled proteomic studies (Pietzner et al., 4775 proteins [10]; Ferkingstad et al., 4719 proteins [11]; Sun_1 et al., 2995 proteins [12]; Sun_2 et al., 1463 proteins [13]; Suhre et al., 1124 proteins [14]; Folkersen et al., 90 proteins [15]; Yao et al., 71 proteins [16]) were employed to extract summary statistics of genetic associations with plasma proteins, among them, six studies [10–12, 14–16] had available full summary-level data. These protein data were measured using the SOMAscan platform in four studies [10–12, 14], the Olink platform in two studies [13, 15], and the xMAP platform in one study [16]. Detailed information on these studies is shown in Additional file 1: Table S2.

### Proteome-wide Mendelian randomization (MR) analysis

The protein quantitative trait loci (pQTLs) from the above-mentioned seven proteomic studies were used for the selection of genetic instruments. The platform ID for each protein from each study was mapped to the gene symbol and unified based on annotations provided by the original studies and manual review (https://biodbnet-abcc.ncifcrf.gov/db/db2db.php). Then, we mapped SNPs to human genome Build 37 (NCBI GRCh37) for unifying genomic coordinates. The following criteria were used to select instruments and proteins: (i) SNPs associated with any protein were selected (*P* < 5×10^−8^); (ii) the SNPs and proteins within the Major Histocompatibility Complex (MHC) region (chr6:25.5–34.0Mb) were excluded due to their complex linkage disequilibrium (LD) structure; (iii) the LD clumping was then conducted to identify independent pQTLs for each protein (*r*^2^ < 0.001); (iv) the *R*^2^ and *F*-statistic (*R*^2^=2×EAF×(1-EAF)×beta^2^; *F*=*R*^2^×(*N*−2)/(1−*R*^2^)) [27] were used to estimate the strength of genetic instruments, where *R*^2^ was the proportion of the variability of the protein levels explained by each genetic instrument. For reduplicative proteins among studies, the protein with the largest sum of *R*^2^ was selected. We further classified instruments as cis or trans pQTLs based on the following criteria: a pQTL was defined as cis pQTL when the leading SNP in the region was located within 1 Mb of the transcription start site of the protein-coding gene, whereas a pQTL lying outside of this region was defined as trans pQTL [12]. Finally, a total of 13,236 instruments (1871 cis pQTLs, 11,377 trans pQTLs) and 4853 unique plasma proteins were included in the analysis. Instrument variables are presented in Additional file 1: Table S3.

The “TwoSampleMR” package [28] was employed to perform MR analysis. For any proteins with only one instrument, the Wald ratio method was used to estimate the log odds change in CRC risk for per standard deviation (SD) increment of circulating protein levels as proxied by the instrumental variables. The inverse-variance weighted (IVW) method was used to obtain the MR effects estimates for proteins with more than one instrument. The heterogeneity test was performed to assess the heterogeneity of the genetic instruments based on the Q statistic. We also performed additional analyses including simple mode, weighted mode, weighted median, and MR-Egger to account for horizontal pleiotropy [29]. MR-Egger results were used only when the intercept indicated the presence of horizontal pleiotropy. Bonferroni correction was used for multiple testing correction, with *P* < 1.03×10^−5^ (0.05/4853) as the significance level. Replication MR analysis was further performed for the identified proteins based on CRC GWAS summary data from FinnGen and UKBB, respectively. *P* value < 0.05 was defined as the significance level for replication. Finally, the estimates for each protein from the CRC meta-GWASs, FinnGen, and UKBB were combined based on the random-effects meta-analysis method. In stratified analysis by tumor site, we further tested associations of the identified protein markers with colon cancer and rectal cancer, respectively. Additionally, we performed a sensitivity analysis using only cis pQTLs as instruments to evaluate associations of proteins with CRC risk, with *P* < 2.70×10^−5^ (0.05/1850 proteins with cis pQTLs) as the significance level. The analyses were conducted using R software 4.1.0.

### Bayesian colocalization analysis

To assess whether two associated signals (protein and CRC risk) were consistent with a shared causal variant to distinguish the confounding of linkage disequilibrium, we employed summary statistics of proteins and CRC meta-GWASs to perform Bayesian colocalization analysis based on the “coloc” package [30]. The colocalization analysis included five hypotheses: (i) there was no causal variant for either protein or CRC in the genomic locus (H0); (ii) there was one causal variant for protein only (H1); (iii) there was one causal variant for CRC only (H2); there were two distinct causal variants for protein and CRC (H3); (iv) there was a shared causal variant for protein and CRC (H4). For each protein, we included SNPs within ±500 kb of the pQTL. When a protein had more than one pQTL, colocalization analysis was performed based on each pQTL, respectively, and the pQTL with the strongest evidence for colocalization was shown. Default parameters were used to perform colocalization, with p1=1×10^−4^ (prior probability a SNP is associated with protein), *p*2=1×10^−4^ (prior probability a SNP is associated with CRC), and p12=1×10^−5^ (prior probability a SNP is associated with both protein and CRC) [30]. Given that colocalization is sensitive to priors and window sizes, we performed additional colocalization analyses based on other priors (p12=1e−6) and windows (±250kb) to evaluate the robustness of the results. The posterior probability was used to quantify the support for each hypothesis. The posterior probability for H4 (PP4) that was higher than 80% under different priors and windows was considered strong evidence of colocalization. The “LocusCompareR” package [31] was used to visualize the region results of colocalization. To further explain the colocalization evidence driven by trans pQTL, we used Reactome (https://reactome.org/) to obtain pathway information of the identified proteins with trans pQTLs and the candidate mapping genes of the trans pQTLs and tested the relationship of mapping gene coding proteins with CRC by colocalization analysis.

### Summary-data-based MR (SMR) analysis

Summary-data-based MR (SMR) analysis was further conducted as a complementary method to verify the causal associations between proteins and CRC [32]. The heterogeneity in dependent instruments (HEIDI) test, using multiple SNPs in a region, was employed to distinguish proteins that were associated with CRC risk owing to a shared genetic variant rather than genetic linkage [32]. The SMR and HEIDI tests were performed using SMR software (SMR v1.3.1) [32]. A *P* value < 3.85×10^−3^ (0.05/13) was defined as the significance level for SMR. The *P* value of the HEIDI test > 0.05 indicated that the association of protein and CRC was not driven by linkage disequilibrium.

After the identification of CRC-related proteins, we conducted a comprehensive literature search and defined proteins that have not been reported to be associated with CRC in either gene polymorphisms, mRNA levels, or protein levels as novel protein markers for CRC.

### Single cell-type expression analysis

The cell type-specific expression of target genes with evidence for a potential causal effect on CRC at the plasma protein levels was further evaluated by employing single-cell RNA-seq data of human colon tumor tissue and adjacent normal tissues profiled from the Gene Expression Omnibus (GEO) from Wang R et al. [33]. The RNA-seq data of colon cancer tumor tissue included 24,871 genes in 1632 cells. Using the “Seurat” package [34], we first carried out data preprocessing and transformation based on the raw single-cell RNA-seq data. The genes with fewer than three counts in one cell and cells with unique feature counts of less than 50 were removed. The NormalizeData and ScaleData functions were then used to normalize and scale the RNA TPM. The “SingleR” package [35] was used to annotate cell types. To examine whether the identified CRC causal protein-coding genes were highly expressed in a particular cell type in colon cancer tumor tissue, the differential expression analysis based on the Wilcoxon Rank Sum test was performed to compare gene expression levels between a cell type and the rest of the other cell types. The genes with an average Log_2_ fold change (Log_2_FC) more than 0.5 and a false discovery rate (FDR) adjusted *P* value less than 0.05 were identified as enrichment genes in a cell type.

### Protein-protein interaction (PPI) and druggability evaluation

To explore the potential interactions between identified proteins, a PPI network was constructed using the STRING database (https://string-db.org/). We further assessed whether the identified proteins can serve as potential therapeutic targets by searching the interactions between these proteins and drugs using DGIdb [36], ChEMBL [37], and DrugBank [38] databases, which prioritized the potential druggable targets by integrating information from drug-gene interactions, gene function, text mining, and expert curation. The information on drug names and the development process of drugs that targeted identified proteins were documented.

## Results

### Proteome-wide MR analysis identified 13 circulating proteins for CRC

**Fig. 2:**
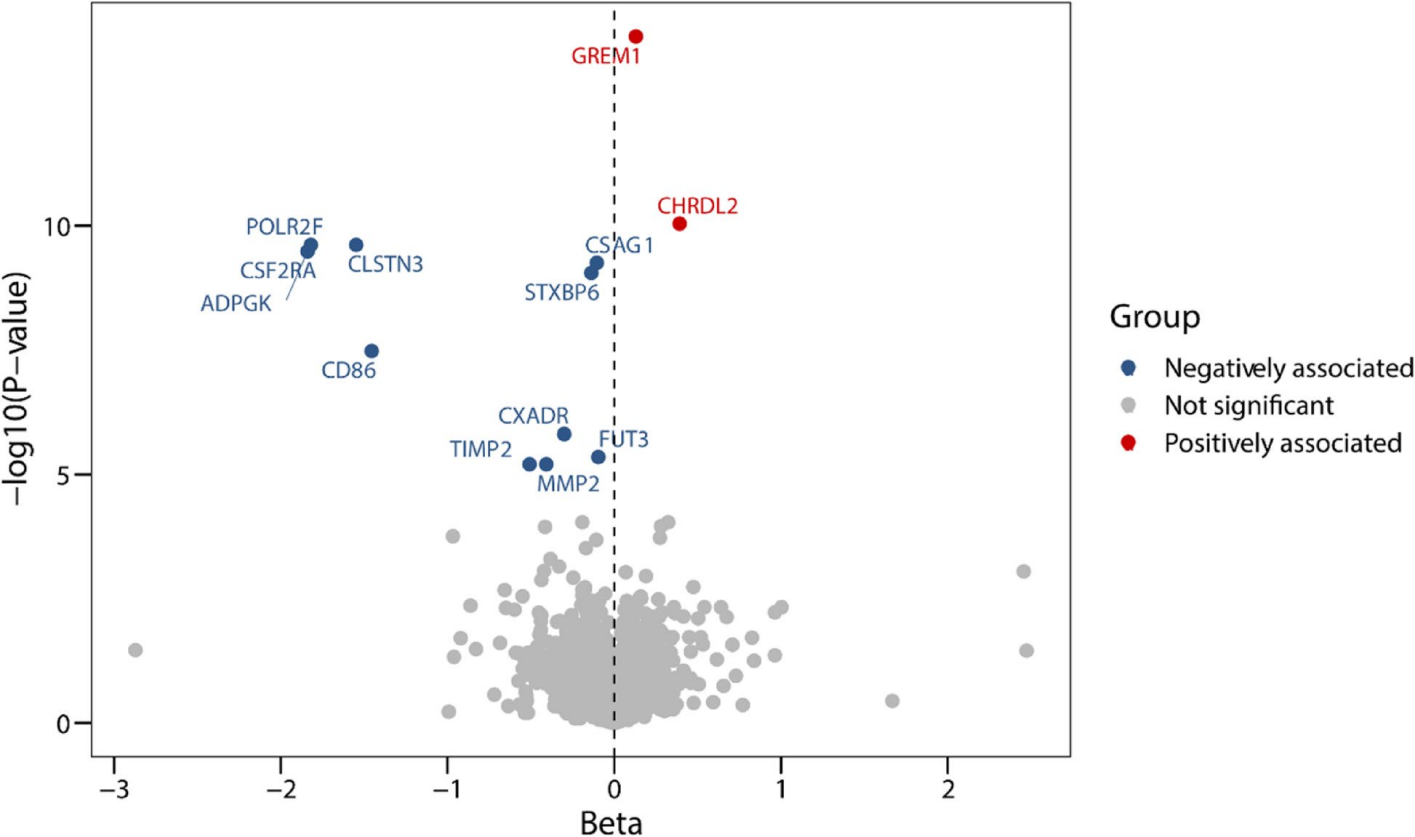
Volcano plot showing results from proteome-wide Mendelian randomization (MR) in the discovery stage

The F-statistics of all genetic instruments were higher than 10, indicating a good strength (Additional file 1: Table S3). Using the Wald ratio or IVW method, a total of 13 proteins were significantly associated with CRC risk after Bonferroni correction (*P* < 1.03×10^−5^) (Table 1 and Fig. 2). Genetically predicted higher levels of GREM1 and CHRDL2 were associated with an increased risk of CRC, while the other 11 proteins (CLSTN3, POLR2F, ADPGK, CSF2RA, CSAG1, STXBP6, CD86, CXADR, FUT3, MMP2, and TIMP2) were negatively associated with CRC risk, suggesting that lower levels of the 11 proteins were associated with a higher risk of CRC. These associations were generally consistent in additional analyses, including weighted mode, weighted median, and MR-Egger, except for simple mode. No heterogeneity and pleiotropy were found (*P*_heterogeneity_ > 0.05, *P*_pleiotropy_ > 0.05) (Additional file 1: Table S4). All results of the discovery proteome-wide MR are shown in Additional file 1: Table S5.

**Table 1:** Summary results from Mendelian randomization (MR), meta, colocalization, and SMR for 13 proteome-wide MR-identified proteins

**Protein**	**Protein full name**	**MR**		**Meta**		**Colocalization** ^b^	**SMR**			**Category**
		*P*_discovery_	*P*_replication_^a^	Beta	*P*	PP4>0.80	Beta	*P*	*P*_HEIDI_	
GREM1	Gremlin-1	1.55E−14	3.72E−09	0.12	2.13E−18	Yes	0.14	1.22E−16	0.27	tier1
CHRDL2	Chordin-like protein 2	8.98E−11	3.54E−05	0.27	3.73E−05	No	0.39	3.35E−08	0.15	tier2
CLSTN3	Calsyntenin-3	2.41E−10	1.59E−06	−1.23	3.27E−09	Yes	−1.63	2.73E−06	0.39	tier1
CSF2RA	Granulocyte-macrophage colony-stimulating factor receptor subunit alpha	3.28E−10	1.04E−06	−1.49	4.82E−10	Yes	−1.69	8.91E−06	0.11	tier1
CD86	T-lymphocyte activation antigen CD86	3.30E−08	1.04E−06	−1.08	9.02E−05	Yes	−1.43	9.11E−07	0.12	tier1
POLR2F	DNA-directed RNA polymerases I, II, and III subunit RPABC2	2.41E−10	1.59E−06	−1.45	3.46E−09	-	-	-	-	tier2
ADPGK	ADP-dependent glucokinase	3.28E−10	1.04E−06	−1.49	4.82E−10	Yes	−1.77	1.14E−05	0.05	tier2
CSAG1	Putative chondrosarcoma-associated gene 1 protein	5.54E−10	7.53E−06	−0.08	4.26E−12	No	−0.1	6.10E−10	0.02	tier3
STXBP6	Syntaxin-binding protein 6	8.94E−10	5.28E−06	−0.11	1.41E−10	No	−0.14	6.20E−10	0.03	tier3
CXADR	Coxsackievirus and adenovirus receptor	1.53E−06	0.21	−0.19	0.02	-	-	-	-	tier2
FUT3	3-galactosyl-N-acetylglucosaminide 4-alpha-L-fucosyltransferase FUT3	4.42E−06	4.36E−04	−0.08	9.88E−09	Yes	−0.1	5.31E−06	0.03	tier2
MMP2	72 kDa type IV collagenase	6.25E−06	0.11	−0.09	0.58	No	−0.41	1.83E−05	0.61	tier3
TIMP2	Metalloproteinase inhibitor 2	6.25E−06	0.11	−0.11	0.58	No	−0.51	3.04E−05	0.54	tier3

*SMR* Summary-data-based Mendelian randomization

^a^The replication MR analysis was performed based on CRC GWAS summary data from FinnGen and UKBB, respectively, but only the most significant *P* value was shown

^b^PP4 values were all higher than 0.80 under different priors (p12=1e−5 or p12=1e−6) and windows (±250kb or ±500kb)

**Fig. 3:**
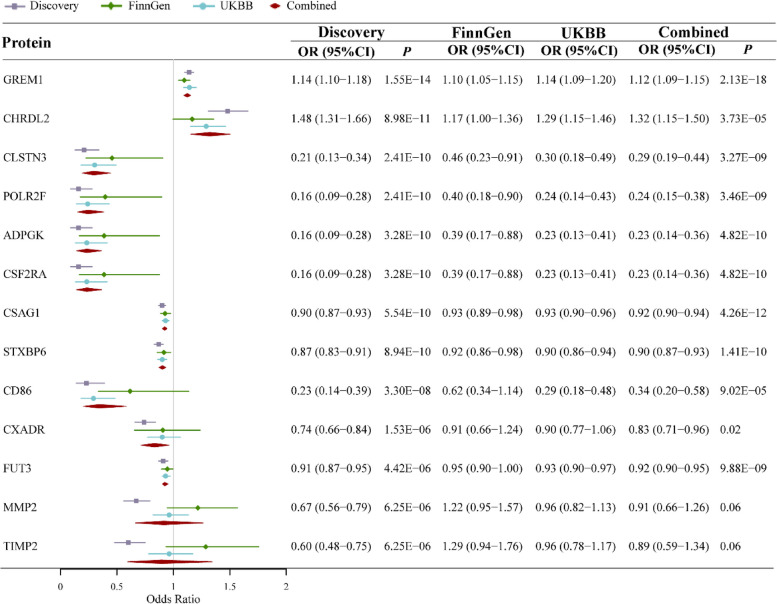
Estimates of meta-analysis from discovery dataset and replication datasets for 13 proteome-wide identified proteins. Discovery dataset: CRC meta-GWASs. Replication datasets: CRC GWAS summary data from FinnGen and UK Biobank (UKBB)

In the replication stage, ten proteins were successfully validated in the FinnGen or the UKBB dataset (*P* < 0.05) based on the Wald ratio or IVW method (Table 1 and Fig. 3). In the meta-analysis of these three sources, 11 proteins showed significant associations, and the odds ratio (OR) (95% confidence interval, CI) of CRC per SD increase in genetically predicted levels of protein was 1.12 (1.09–1.15) for GREM1, 1.32 (1.15–1.50) for CHRDL2, whereas 0.29 (0.19–0.44) for CLSTN3, 0.24 (0.15–0.38) for POLR2F, 0.23 (0.14–0.36) for ADPGK, 0.23 (0.14–0.36) for CSF2RA, 0.92 (0.90–0.94) for CSAG1, 0.90 (0.87–0.93) for STXBP6, 0.34 (0.20–0.58) for CD86, 0.83 (0.71–0.96) for CXADR, and 0.92 (0.90–0.95) for FUT3 (Fig. 3).

In stratified analysis by tumor site (Additional file 1: Tables S6 and S7), eight (STXBP6, CSAG1, CD86, POLR2F, CLSTN3, ADPGK, CSF2RA, CHRDL2) of the 13 proteins were associated with colon cancer risk using Wald ratio or IVW method, and two (CHRDL2, CD86) were associated with rectal cancer risk (*P* < 0.05). Among them, CHRDL2 and CD86 were associated with both colon and rectal cancers, with consistent direction. No heterogeneity and pleiotropy were found (*P*_heterogeneity_ > 0.05, *P*_pleiotropy_ > 0.05).

In sensitivity analysis (cis only MR), four of 13 proteins had cis pQTLs, and three (GREM1, CHRDL2, FUT3) of them were still significantly associated with CRC risk (*P* < 2.70×10^−5^) based on Wald ratio or IVW method, with a consistent direction with the primary analysis (cis+trans MR). Additionally, three other proteins (LAMB1_LAMC1_LAMA1, CABLES2, KLK1) were also found to be significantly associated with CRC risk (*P* < 2.70×10^−5^) in cis only MR (Additional file 1: Table S8).

### Colocalization analysis supported the causality of six proteins with CRC

Of the 13 potential causal proteins identified by proteome-wide MR, two proteins (POLR2F and CXADR) did not have complete summary-level data available and therefore could not be tested by colocalization analysis. Six of the other 11 proteins (GREM1 and FUT3 with cis pQTL; CLSTN3, CSF2RA, CD86, and ADPGK with trans pQTL) were supported by strong evidence of genetic colocalization (PP4 > 80%) under different priors and windows (Table 1, Additional file 1: Table S9), indicating high probability for a shared causal variant between protein level and CRC risk. CSF2RA, CD86, and their trans pQTLs mapping gene (SH2B3) were involved in the same biological pathways (Additional file 1: Table S9). The same biological pathways were not found between CLSTN3 and its trans pQTL mapping gene (ATXN2) and ADPGK and its trans pQTL mapping gene (SH2B3), and no colocalization evidence between SH2B3 and CRC was found. Additional file 2: Fig. S1–S11 show the regional association for colocalization results.

### SMR and HEIDI tests verified seven causal proteins

To further verify the observed findings, we performed SMR and HEIDI tests for 11 proteins with full summary-level data. All of 11 proteins passed the SMR test (*P* < 3.85×10^−3^), and seven of them passed the HEIDI test (*P* > 0.05) (Table 1). The SMR locus plot and effect plots of seven proteins are shown in Additional file 2: Fig. S12–S18. Combining the above evidence, we classified these proteins into three tiers. Four proteins (GREM1, CLSTN3, CSF2RA, CD86) passed all tests and were classified into tier 1 (Table 1). Five proteins that failed colocalization analysis or HEIDI test or that were not able to be tested due to the lack of data (CHRDL2, POLR2F, ADPGK, CXADR, FUT3) were classified into tier 2. Four proteins (CSAG1, STXBP6, MMP2, TIMP2) failed in the replication MR and meta-analysis or failed in both colocalization analysis and HEIDI test were classified into tier 3.

### Cell-type specificity expression in the colon tumor tissue

**Fig. 4:**
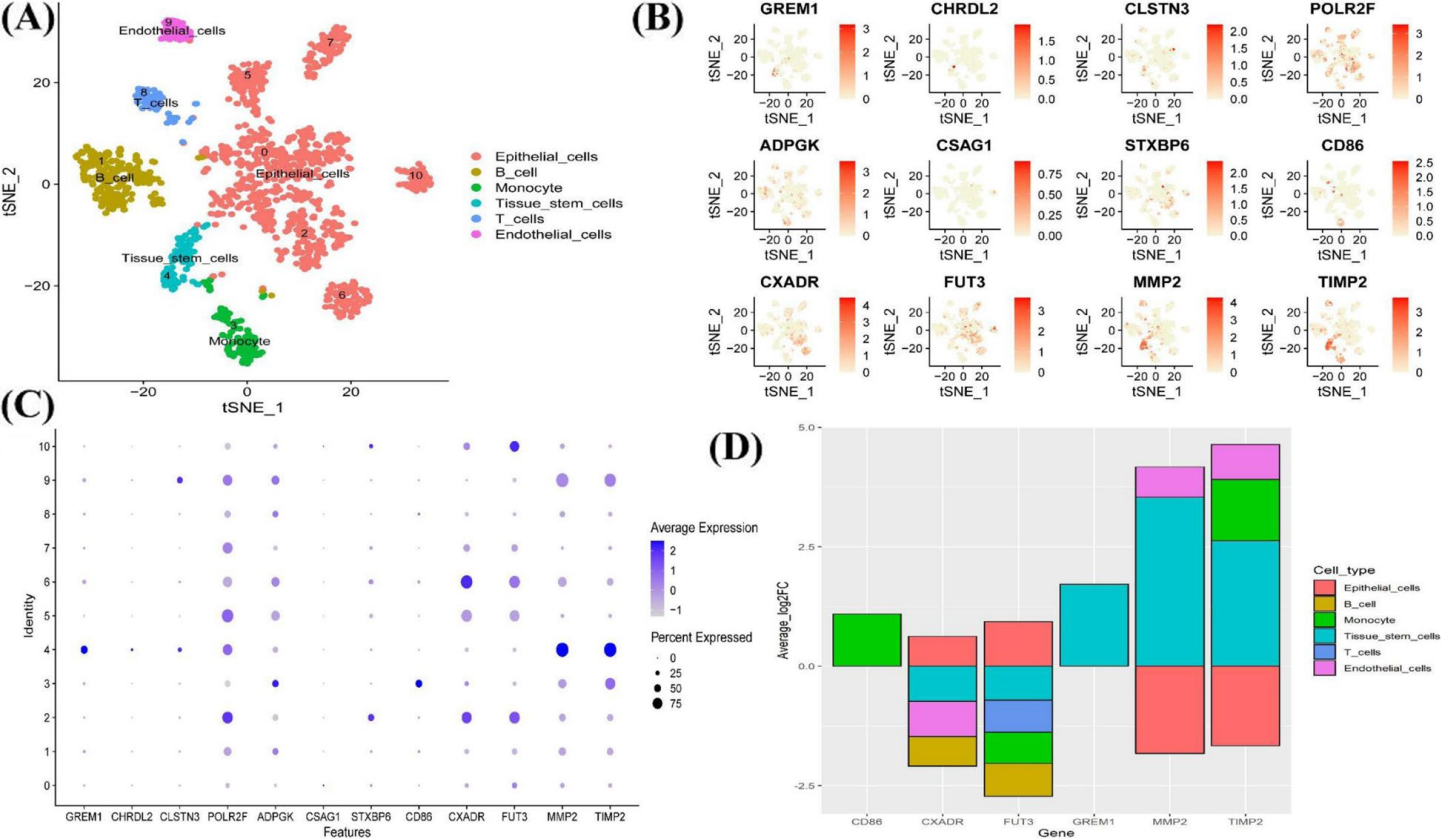
Single-cell type expression in colon tumor tissue for the coding genes of proteins identified by proteome-wide Mendelian randomization. **A** A total of 11 cell clusters and six cell types were identified. **B** and **C** show the expression of protein coding genes in each cluster. **D** Six protein-coding genes had evidence of enrichment in a cell type at average Log_2_FC > 0.5 and FDR < 0.05 level

To explore whether the coding genes of 13 circulating proteins had any cell type-specific enrichment in colon tumor tissue, we further performed single cell-type expression analysis using single-cell RNA-seq data from GEO. Cells were clustered into 11 clusters and were further classified into six cell types (epithelial cells, B cell, monocyte, tissue stem cells, T cells, endothelial cells) (Fig. 4A). 12 of the 13 protein-coding genes had expression data in colon tumor tissue, whereas *CSF2RA* expression was undetected; Fig. 4 (B and C) shows single-cell expression of these 12 coding genes in every cluster. Among them, six protein-coding genes had cell type-specific enrichment in colon tumor tissue at average Log_2_FC > 0.5 and FDR < 0.05 level (Fig. 4D). *GREM1*, *MMP2*, and *TIMP2* were mainly enriched in tissue stem cells, whereas *FUT3* and *CXADR* were enriched in epithelial cells, and *CD86* was enriched in monocyte. In normal colon tissue, 10 of the 13 protein-coding genes had expression data, whereas the expression of *CHRDL2*, *CSF2RA*, and *CSAG1* was undetected. Five protein-coding genes had cell type-specific enrichment in normal colon tissue at average Log_2_FC > 0.5 and FDR < 0.05 level: *CD86* and *TIMP2* were mainly enriched in dendritic cell (DC), whereas *MMP2* was enriched in fibroblasts, and *FUT3* was enriched in epithelial cells (Additional file 2: Fig. S19).

### PPI and druggability evaluation on the potentials of therapeutic targets

The protein-protein interaction analysis found limited interactions between the identified potential causal proteins, and only the interaction between GREM1 and CHRDL2, and the interaction between MMP2 and TIMP2 were identified (Additional file 2: Fig. S20) which were involved in osteoclast differentiation and tumorigenesis pathways, respectively. In druggability evaluation, we found that four of these proteins (POLR2F, CSF2RA, CD86, MMP2) have been targeted for drug development (Additional file 1: Table S10). Drug (TAS-106) targeting POLR2F has been found to inhibit the growth of colorectal and gastric tumors in mice. Some drugs targeting CSF2RA have been developed to treat autoimmune diseases (sargramostim, KB002), diabetic foot ulcers (foreskin fibroblast), and accelerate wound closure and healing (foreskin keratinocyte). Among them, clove oil with antioxidant and antimicrobial activity has been categorized as generally recognized as safe (GRAS) as a food additive or for use in dental cement by the Food and Drug Administration (FDA). Some drugs targeting CD86 have been approved for the treatment of rheumatoid arthritis (abatacept, belatacept) and acute graft-versus-host disease (abatacept, belatacept, antithymocyte immunoglobulin). Drugs targeting MMP2 have been used for the treatment of cancer (marimastat), renovascular hypertension, and congestive heart failure (captopril), or investigated in clinical trials for the treatment of lung cancer (oleandrin).

## Discussion

In this study, we present a comprehensive investigation on the causal associations between 4853 plasma proteins and CRC risk. The discovery proteome-wide MR identified 13 protein markers, among which genetically determined higher levels of two proteins and lower levels of 11 proteins were associated with increased CRC susceptibility. Two proteins were significantly associated with both colon and rectal cancers in stratified analysis by tumor site. The replication MR and meta-analysis validated 11 of these 13 candidate proteins. Bayesian colocalization highlighted the causal effects of six protein biomarkers, and seven proteins were verified by SMR and HEIDI tests. Collectively, we identified four proteins (GREM1, CLSTN3, CSF2RA, CD86) with the most convincing evidence (tier 1), five proteins with convincing evidence (tier 2), and four proteins with middle convincing evidence (tier 3), among which six (CLSTN3, POLR2F, ADPGK, CSAG1, STXBP6, FUT3) were novel plasma protein makers associated with CRC. We further verified the differential expressions of these protein-coding genes in the tissue stem cells, epithelia, and monocytes. Druggability evaluation prioritized four protein biomarkers, which have been developed as drug targets for autoimmune diseases and cancer, with the potentials of being repurposed as therapeutic targets for CRC.

Our analysis implicated candidate proteins that have been reported evidence with CRC in either gene polymorphisms, mRNA levels, or protein levels from previous genetic or experiment studies, including GREM1, CHRDL2, CSF2RA, CD86, CXADR, MMP2, and TIMP2, among which three (GREM1, CSF2RA, CD86) were prioritized with the most convincing evidence (tier 1). GREM1 (Gremlin-1) acts as an antagonist of bone morphogenic protein (BMP), and BMP is closely involved in the development of CRC [39]. *GREM1* gene polymorphisms have been observed to be associated with CRC risk in multiple ethnic groups [40], and a higher expression of *GREM1* was associated with poor survival in CRC patients [41]. Experiment studies have also shown that the overexpression of *GREM1* led to colonic tumorigenesis [42]. In line with these findings, we expanded the evidence and confirmed the causal role of elevated GREM1 protein levels in CRC risk. Although lack of drug information targeting GREM1, studies have been found that specific anti-GREM1 therapeutic antibody has a strong tumor-inhibitory effect on prostate cancer [43] and CRC tumoroid [41]. CSF2RA (Granulocyte-macrophage colony-stimulating factor receptor subunit alpha, known as GM-CSF) is a growth factor with biological functions of mediating inflammation and pain. Laboratory experiments showed a significant inhibiting effect of GM-CSF-stimulated macrophages on the proliferation of CRC cells, and GM-CSF production by CRC cells was related to improved survival [44]. Consistently, we expanded the evidence from the population and confirmed the causal effect of reduced CSF2RA protein levels on CRC risk. Drug targeting CSF2RA, such as sargramostim, has shown clinical activity against autoimmune diseases and diverse solid tumors [45]. CD86 (T-lymphocyte activation antigen CD86) is the costimulatory molecule on antigen-presenting cells, playing an important role in autoimmunity and tumor immunity. *CD86* gene polymorphism has been linked to CRC risk in multiple populations [46]. The CD86 protein level was negatively associated with the CRC tumor differentiation and tumor node metastasis (TNM) stage, and was related to improved survival [47].

We additionally found several novel candidate proteins for CRC, including CLSTN3, POLR2F, ADPGK, CSAG1, STXBP6, and FUT3, among which CLSTN3 was prioritized with the most convincing evidence (tier 1). CLSTN3 (Calsyntenin-3), localizing to the postsynaptic membrane, serves as a synaptogenic adhesion molecule and can trigger presynaptic differentiation. *CLSTN3* gene polymorphism led to dysfunction in white adipose tissue [48] and was associated with obesity that was closely related to CRC risk. Although direct evidence on CLSTN3 protein and CRC risk is unreported, evidence from the human protein atlas has shown that high expression of *CLSTN3* is favorable for prognostic of pancreatic cancer, breast cancer, and urothelial cancer [49]. Further epidemiological studies and experimental researches are needed to ascertain our findings.

The strength of this study is that we systematically examined the associations between plasma protein biomarkers and CRC risk by employing a two-stage proteome-wide MR design with the advantages of large sample sizes, rich proteome coverage, and minimal risk of reverse causation and confounding bias. The consistency of results among multiple rigorous analyses confirmed the robustness of the study findings. Additional evidence from single cell-type expression analysis, PPI, and druggability evaluation provided insights into the potential pathogenic effect of candidate proteins on CRC and further prioritized druggable targets. Although the lack of drug information of several proteins (e.g., GREM1 and CHRDL2), these proteins still deserve to be a promising new therapeutic target for CRC. In particular, GREM1 has been found that anti-GREM1 therapeutic antibody has a strong tumor-inhibitory effect on prostate cancer [43] and CRC tumoroid [41]. Nevertheless, several limitations of this study should also be considered. First, the current analysis was restricted to European populations. The generalization of these findings to other ancestries needs to be further confirmed. However, several candidate biomarkers have also previously been reported to be linked to CRC as gene polymorphisms, mRNA levels, or protein levels based on different ethnic groups, which may imply a degree of generalization between ancestries. Second, we assessed the role of plasma proteins in CRC but could not estimate the levels of relevant proteins in other tissues. Assessing the role of protein levels from other tissues in CRC may provide more insight into CRC pathogenesis, especially intestinal tissue. Third, the strict significance threshold and evidence grading criteria may lead to underestimation the convincing of the associated proteins, such as POLR2F, which could not be tested by colocalization and SMR due to the lack of full summary-level data. Furthermore, the current statistical analyses and strict significance threshold might filter out these plasma proteins that are “downstream” of the “driver” proteins. Further mechanistic studies are needed to uncover the “driver” and “downstream” proteins involved in CRC onset and development. Fourth, 62% of protein markers had only trans pQTLs. Although trans pQTLs can help to expand the understanding of the relationship between proteins, diseases, and the etiology of diseases [12, 50], the interpretation of the current findings is difficult. This is due to insufficient biological understanding of trans pQTLs and proteins, which does not allow to ascertain causality with CRC. Nevertheless, some proteins with trans pQTLs (e.g., CSF2RA, CD86) had robust colocalization evidence and shared the same biological pathways between them and their trans pQTLs mapping gene, indicating potential vertical pleiotropy. Additionally, 1.4% of proteins (e.g., LAMB1_LAMC1_LAMA1) are unable to be distinguished by the current assay, so it is difficult to ascertain the specific relationships of them with CRC. Lastly, plasma protein may also be affected by factors other than genetics. In the current study, the protein levels explained by independent genetic instruments (R^2^_sum) ranged from 0.09% to 82.54%, and future epidemiological studies of measured plasma protein levels and CRC risk are needed to validate the findings.

## Conclusions

Our study identified several plasma proteins that were associated with CRC risk and provided new insights into the etiology of CRC and promising targets for the development of screening biomarkers and therapeutic drugs for CRC. Further experimental and clinical studies are needed to evaluate the utility and efficacy of these candidates to ascertain the current findings.

### Supplementary Information

**Additional file 1: Table S1.** Summary of colorectal cancer (CRC), colon cancer, and rectal cancer datasets used in the current study. **Table S2.** Summary of proteins datasets used in the current study. **Table S3.** Genetic instruments for plasma proteins. **Table S4.** Results of 13 proteome-wide Mendelian randomization identified proteins for colorectal cancer risk. **Table S5.** All results of discovery proteome-wide Mendelian randomization for colorectal cancer risk. **Table S6.** Mendelian randomization results of 13 proteins with colon cancer risk. **Table S7.** Mendelian randomization results of 13 proteins with rectal cancer risk. **Table S8.** All results of proteome-wide Mendelian randomization for colorectal cancer risk using only cis pQTLs. **Table S9:** Colocalization results across different priors and windows. **Table S10:** Druggability of proteins potentially causally associated with colorectal cancer.

**Additional file 2: Fig S1.** Regional association plot for colocalization analysis of GREM1 protein with colorectal cancer (CRC) risk. **Fig S2.** Regional association plot for colocalization analysis of CHRDL2 protein with colorectal cancer risk. **Fig S3.** Regional association plot for colocalization analysis of CLSTN3 protein with colorectal cancer risk. **Fig S4.** Regional association plot for colocalization analysis of ADPGK protein with colorectal cancer risk. **Fig S5.** Regional association plot for colocalization analysis of CSF2RA protein with colorectal cancer risk. **Fig S6.** Regional association plot for colocalization analysis of CSAG1 protein with colorectal cancer risk. **Fig S7.** Regional association plot for colocalization analysis of STXBP6 protein with colorectal cancer risk. **Fig S8.** Regional association plot for colocalization analysis of CD86 protein with colorectal cancer risk. **Fig S9.** Regional association plot for colocalization analysis of FUT3 protein with colorectal cancer risk. **Fig S10.** Regional association plot for colocalization analysis of MMP2 protein with colorectal cancer risk. **Fig S11.** Regional association plot for colocalization analysis of TIMP2 protein with colorectal cancer risk. **Fig S12.** The summary-data-based Mendelian randomization (SMR) result of GREM1 with colorectal cancer (CRC) risk. **Fig S13.** The summary-data-based Mendelian randomization (SMR) result of CHRDL2 with colorectal cancer (CRC) risk. **Fig S14.** The summary-data-based Mendelian randomization (SMR) result of CLSTN3 with colorectal cancer (CRC) risk. **Fig S15.** The summary-data-based Mendelian randomization (SMR) result of CSF2RA with colorectal cancer (CRC) risk. **Fig S16.** The summary-data-based Mendelian randomization (SMR) result of CD86 with colorectal cancer (CRC) risk. **Fig S17.** The summary-data-based Mendelian randomization (SMR) result of MMP2 with colorectal cancer (CRC) risk. **Fig S18.** The summary-data-based Mendelian randomization (SMR) result of TIMP2 with colorectal cancer (CRC) risk. **Fig S19.** Single-cell type expression in normal colon tissue for the coding genes of proteins identified by proteome-wide Mendelian randomization. **Fig S20.** The Protein-protein interaction (PPI) network of proteins identified by proteome-wide Mendelian randomization.

## Data Availability

The results of this study are included in this published article and its supplementary information files. The UK Biobank is an open access resource and bona fide researchers can apply to use the UK Biobank dataset by registering and applying at http://ukbiobank.ac.uk/register-apply/ [25]. The GWAS summary data of FinnGen are available at https://www.finngen.fi/en/access_results [24]. The GWAS summary data of colon cancer and rectal cancer are available at https://github.com/Wittelab/pancancer_pleiotropy [26]. The GWAS summary data of proteins are available at https://omicscience.org/apps/pgwas/ [10], https://www.decode.com/summarydata/ [11], http://www.phpc.cam.ac.uk/ceu/proteins/ [12], http://proteomics.gwas.eu [14], https://preview.ncbi.nlm.nih.gov/gap/eqtl/studies/ [16], and https://doi.org/10.5281/zenodo.2615265 [15]. The single-cell RNA-seq data of human colon tumor tissue and adjacent normal tissues are available at the Gene Expression Omnibus (GEO), https://www.ncbi.nlm.nih.gov/geo/query/acc.cgi?acc=GSE110009 [33].

## Abbreviations

CRC: Colorectal cancer
FDR: False discovery rate
GEO: Gene Expression Omnibus
GWASs: Genome-wide association studies
HEIDI: Heterogeneity in dependent instruments
IVW: Inverse-variance weighted
Log_2_FC: Log_2_ fold change
MR: Mendelian randomization
PPI: Protein-protein interaction
pQTLs: Protein quantitative trait loci
SD: Standard deviation
SMR: Summary-data-based Mendelian randomization
