# Supplementary material for: Identification of novel protein biomarkers and drug targets for colorectal cancer by integrating human plasma proteome with genome
Source: Genome Med. 2023 Sep 19;15:75. doi: 10.1186/s13073-023-01229-9 (PMC10508028; doi:10.1186/s13073-023-01229-9)
Supplement: Supplementary file 2 — Additional file 2: Fig S1. Regional association plot for colocalization analysis of GREM1 protein with colorectal cancer (CRC) risk. Fig S2. Regional association plot for colocalization analysis of CHRDL2 protein with colorectal cancer risk. Fig S3. Regional association plot for colocalization analysis of CLSTN3 protein with colorectal cancer risk. Fig S4. Regional association plot for colocalization analysis of ADPGK protein with colorectal cancer risk. Fig S5. Regional association plot for colocalization analysis of CSF2RA protein with colorectal cancer risk. Fig S6. Regional association plot for colocalization analysis of CSAG1 protein with colorectal cancer risk. Fig S7. Regional association plot for colocalization analysis of STXBP6 protein with colorectal cancer risk. Fig S8. Regional association plot for colocalization analysis of CD86 protein with colorectal cancer risk. Fig S9. Regional association plot for colocalization analysis of FUT3 protein with colorectal cancer risk. Fig S10. Regional association plot for colocalization analysis of MMP2 protein with colorectal cancer risk. Fig S11. Regional association plot for colocalization analysis of TIMP2 protein with colorectal cancer risk. Fig S12. The summary-data-based Mendelian randomization (SMR) result of GREM1 with colorectal cancer (CRC) risk. Fig S13. The summary-data-based Mendelian randomization (SMR) result of CHRDL2 with colorectal cancer (CRC) risk. Fig S14. The summary-data-based Mendelian randomization (SMR) result of CLSTN3 with colorectal cancer (CRC) risk. Fig S15. The summary-data-based Mendelian randomization (SMR) result of CSF2RA with colorectal cancer (CRC) risk. Fig S16. The summary-data-based Mendelian randomization (SMR) result of CD86 with colorectal cancer (CRC) risk. Fig S17. The summary-data-based Mendelian randomization (SMR) result of MMP2 with colorectal cancer (CRC) risk. Fig S18. The summary-data-based Mendelian randomization (SMR) result of TIMP2 with colorectal cancer (CRC [file 13073_2023_1229_MOESM2_ESM.pdf]

Supplementary Figures for

Identification of novel protein biomarkers and drug targets for colorectal cancer by integrating human plasma proteome with genome

Fig S1 Regional association plot for colocalization analysis of GREM1 protein with colorectal cancer (CRC) risk. The lead SNP is shown as a purple diamond. SNPs within ±500 kb of the protein quantitative trait locus were included; p12=1e-5, prior probability a SNP is associated with both protein and CRC.

Fig S2 Regional association plot for colocalization analysis of CHRDL2 protein with colorectal cancer risk. The lead SNP is shown as a purple diamond. SNPs within ±500 kb of the protein quantitative trait locus were included; p12=1e-5, prior probability a SNP is associated with both protein and CRC.

Fig S3 Regional association plot for colocalization analysis of CLSTN3 protein with colorectal cancer risk. The lead SNP is shown as a purple diamond. SNPs within ±500 kb of the protein quantitative trait locus were included; p12=1e-5, prior probability a SNP is associated with both protein and CRC.

Fig S4 Regional association plot for colocalization analysis of ADPGK protein with colorectal cancer risk. The lead SNP is shown as a purple diamond. SNPs within ±500 kb of the protein quantitative trait locus were included; p12=1e-5, prior probability a SNP is associated with both protein and CRC.

Fig S5 Regional association plot for colocalization analysis of CSF2RA protein with colorectal cancer risk. The lead SNP is shown as a purple diamond. SNPs within ±500 kb of the protein quantitative trait locus were included; p12=1e-5, prior probability a SNP is associated with both protein and CRC.

Fig S6 Regional association plot for colocalization analysis of CSAG1 protein with colorectal cancer risk. The lead SNP is shown as a purple diamond. SNPs within ±500 kb of the protein quantitative trait locus were included; p12=1e-5, prior probability a SNP is associated with both protein and CRC.

Fig S7 Regional association plot for colocalization analysis of STXBP6 protein with colorectal cancer risk. The lead SNP is shown as a purple diamond. SNPs within ±500 kb of the protein quantitative trait locus were included; p12=1e-5, prior probability a SNP is associated with both protein and CRC.

Fig S8 Regional association plot for colocalization analysis of CD86 protein with colorectal cancer risk. The lead SNP is shown as a purple diamond. SNPs within ±500 kb of the protein quantitative trait locus were included; p12=1e-5, prior probability a SNP is associated with both protein and CRC.

Fig S9 Regional association plot for colocalization analysis of FUT3 protein with colorectal cancer

risk. The lead SNP is shown as a purple diamond. SNPs within ±500 kb of the protein quantitative trait locus were included; p12=1e-5, prior probability a SNP is associated with both protein and CRC.

Fig S10 Regional association plot for colocalization analysis of MMP2 protein with colorectal cancer risk. The lead SNP is shown as a purple diamond. SNPs within ±500 kb of the protein quantitative trait locus were included; p12=1e-5, prior probability a SNP is associated with both protein and CRC.

Fig S11 Regional association plot for colocalization analysis of TIMP2 protein with colorectal cancer risk. The lead SNP is shown as a purple diamond. SNPs within ±500 kb of the protein quantitative trait locus were included; p12=1e-5, prior probability a SNP is associated with both protein and CRC.

Fig S12 The summary-data-based Mendelian randomization (SMR) result of GREM1 with colorectal cancer (CRC) risk. (A) The SMR locus plot. Top plot, gray dots represent the *P* values for SNPs from the CRC meta-GWASs, diamond represents the *P* value for GREM1 from the SMR test. Bottom plot shows the summary statistics of GREM1 protein. (B) The SMR effect plot. The horizontal axis represents the effect sizes of SNPs on GREM1, while the vertical axis represents the effect sizes of SNPs on CRC risk. pOTL, protein quantitative trait locus.

Fig S13 The summary-data-based Mendelian randomization (SMR) result of CHRDL2 with colorectal cancer (CRC) risk. (A) The SMR locus plot. Top plot, gray dots represent the *P* values for SNPs from the CRC meta-GWASs, diamond represents the *P* value for CHRDL2 from the SMR test. Bottom plot shows the summary statistics of CHRDL2 protein. (B) The SMR effect plot. The horizontal axis represents the effect sizes of SNPs on CHRDL2, while the vertical axis represents the effect sizes of SNPs on CRC risk. pOTL, protein quantitative trait locus.

Fig S14 The summary-data-based Mendelian randomization (SMR) result of CLSTN3 with colorectal cancer (CRC) risk. (A) The SMR locus plot. Top plot, gray dots represent the *P* values for SNPs from the CRC meta-GWASs, diamond represents the *P* value for CLSTN3 from the SMR test. Bottom plot shows the summary statistics of CLSTN3 protein. (B) The SMR effect plot. The horizontal axis represents the effect sizes of SNPs on CLSTN3, while the vertical axis represents the effect sizes of SNPs on CRC risk. pOTL, protein quantitative trait locus.

Fig S15 The summary-data-based Mendelian randomization (SMR) result of CSF2RA with colorectal cancer (CRC) risk. (A) The SMR locus plot. Top plot, gray dots represent the *P* values for SNPs from the CRC meta-GWASs, diamond represents the *P* value for CSF2RA from the SMR test. Bottom plot shows the summary statistics of CSF2RA protein. (B) The SMR effect plot. The horizontal axis represents the effect sizes of SNPs on CSF2RA, while the vertical axis represents the effect sizes of SNPs on CRC risk. pOTL, protein quantitative trait locus.

Fig S16 The summary-data-based Mendelian randomization (SMR) result of CD86 with colorectal cancer (CRC) risk. (A) The SMR locus plot. Top plot, gray dots represent the *P* values for SNPs

from the CRC meta-GWASs, diamond represents the *P* value for CD86 from the SMR test. Bottom plot shows the summary statistics of CD86 protein. (B) The SMR effect plot. The horizontal axis represents the effect sizes of SNPs on CD86, while the vertical axis represents the effect sizes of SNPs on CRC risk. pOTL, protein quantitative trait locus.

Fig S17 The summary-data-based Mendelian randomization (SMR) result of MMP2 with colorectal cancer (CRC) risk. (A) The SMR locus plot. Top plot, gray dots represent the *P* values for SNPs from the CRC meta-GWASs, diamond represents the *P* value for MMP2 from the SMR test. Bottom plot shows the summary statistics of MMP2 protein. (B) The SMR effect plot. The horizontal axis represents the effect sizes of SNPs on MMP2, while the vertical axis represents the effect sizes of SNPs on CRC risk. pOTL, protein quantitative trait locus.

Fig S18 The summary-data-based Mendelian randomization (SMR) result of TIMP2 with colorectal cancer (CRC) risk. (A) The SMR locus plot. Top plot, gray dots represent the *P* values for SNPs from the CRC meta-GWASs, diamond represents the *P* value for TIMP2 from the SMR test. Bottom plot shows the summary statistics of TIMP2 protein. (B) The SMR effect plot. The horizontal axis represents the effect sizes of SNPs on TIMP2, while the vertical axis represents the effect sizes of SNPs on CRC risk. pOTL, protein quantitative trait locus.

Fig S19 Single-cell type expression in normal colon tissue for the coding genes of proteins identified by proteome-wide Mendelian randomization. (A) A total of nine cell clusters and four cell types were identified. (B) and (C) show the expression of protein coding genes in each cluster. (D) Five protein-coding genes had evidence of enrichment in a cell type at average $Log_2FC > 0.5$ and $FDR < 0.05$ level. DC, dendritic cell.

Fig S20 The Protein-protein interaction (PPI) network of proteins identified by proteome-wide Mendelian randomization. Lines represent interactions between proteins. Green line indicates gene neighborhood and predicted interaction; Blue line indicates known interaction from curated databases; Fuchsia line indicates known interaction that is experimentally determined. Black line indicates co-expression. Data information was from STRING database.

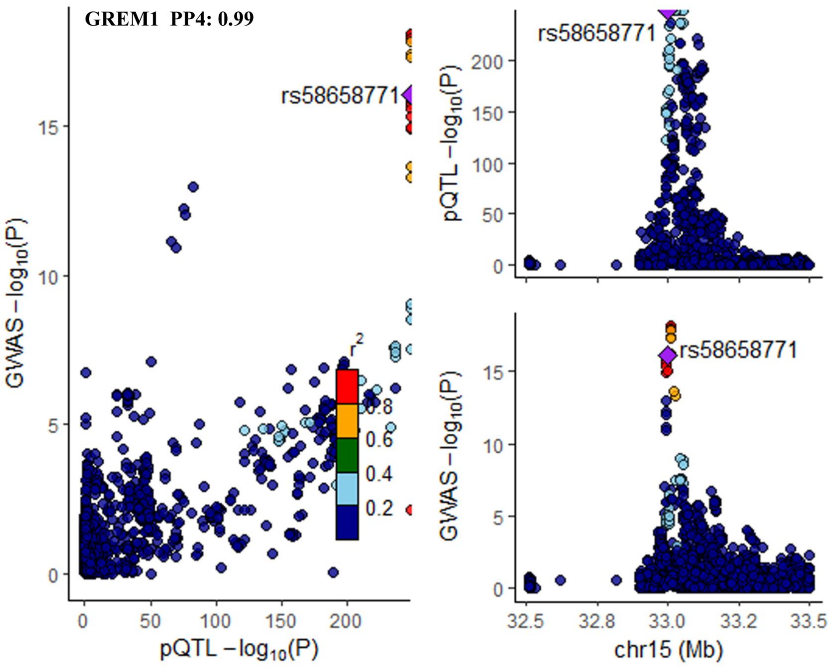

Fig S1 Regional association plot for colocalization analysis of GREM1 protein with colorectal cancer risk. The lead SNP is shown as a purple diamond. SNPs within ±500 kb of the protein quantitative trait locus were included; p12=1e-5, prior probability a SNP is associated with both protein and CRC.

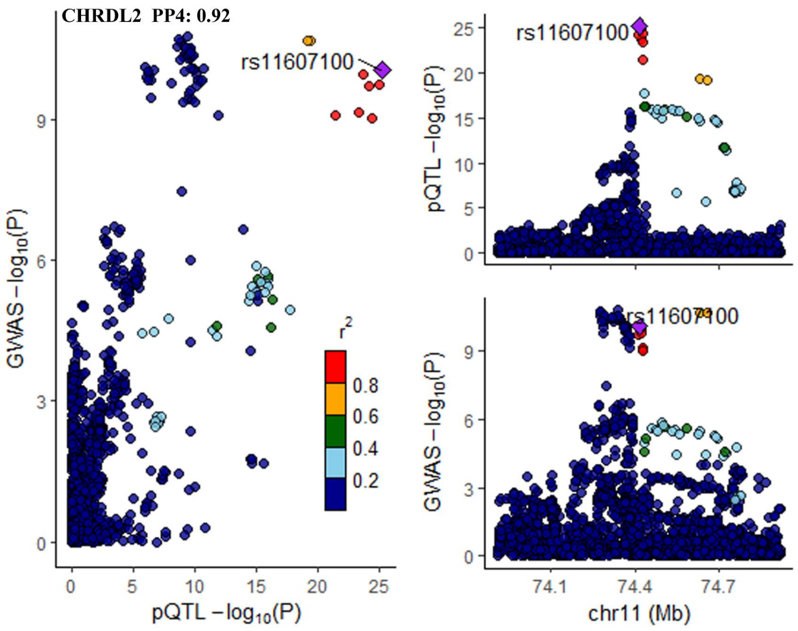

Fig S2 Regional association plot for colocalization analysis of CHRDL2 protein with colorectal cancer risk. The lead SNP is shown as a purple diamond. SNPs within ±500 kb of the protein quantitative trait locus were included; p12=1e-5, prior probability a SNP is associated with both protein and CRC.

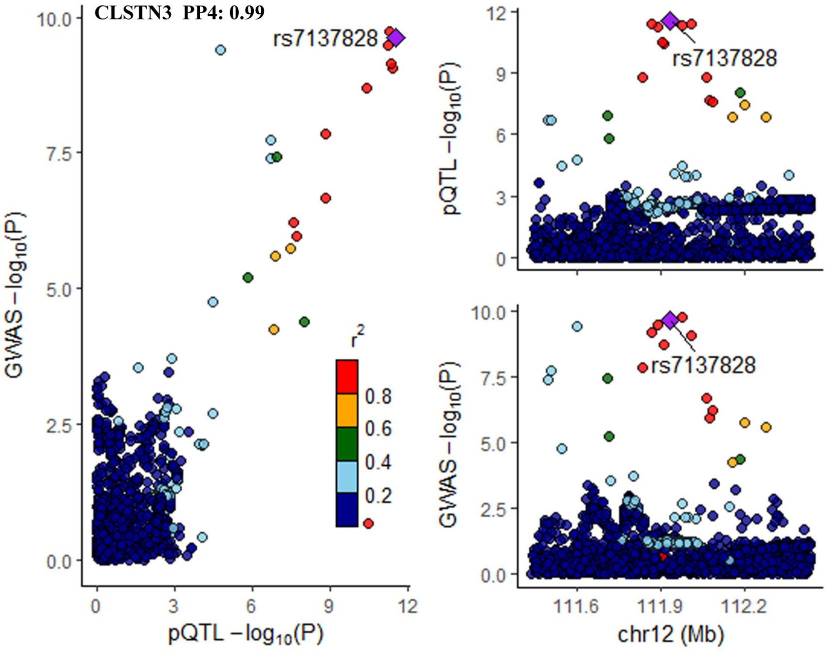

Fig S3 Regional association plot for colocalization analysis of CLSTN3 protein with colorectal cancer risk. The lead SNP is shown as a purple diamond. SNPs within ±500 kb of the protein quantitative trait locus were included; p12=1e-5, prior probability a SNP is associated with both protein and CRC.

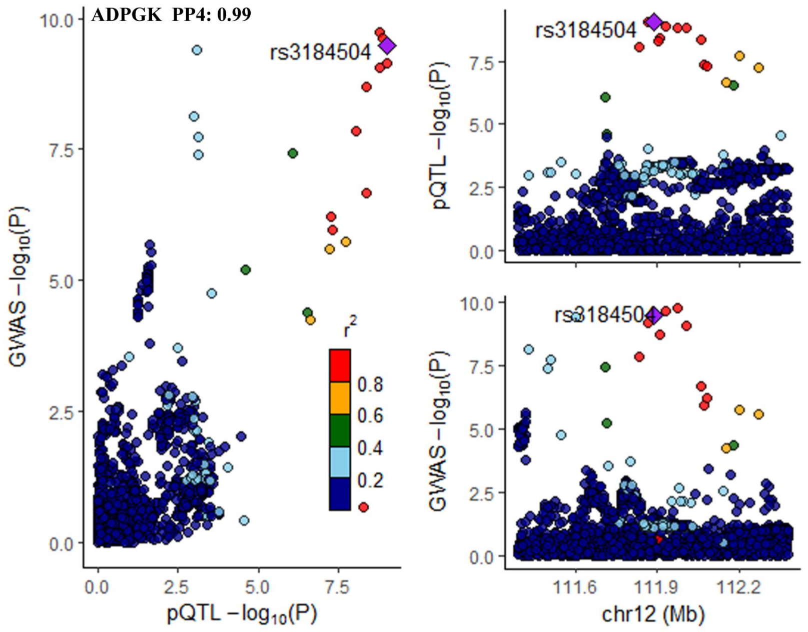

Fig S4 Regional association plot for colocalization analysis of ADPGK protein with colorectal cancer risk. The lead SNP is shown as a purple diamond. SNPs within ±500 kb of the protein quantitative trait locus were included; p12=1e-5, prior probability a SNP is associated with both protein and CRC.

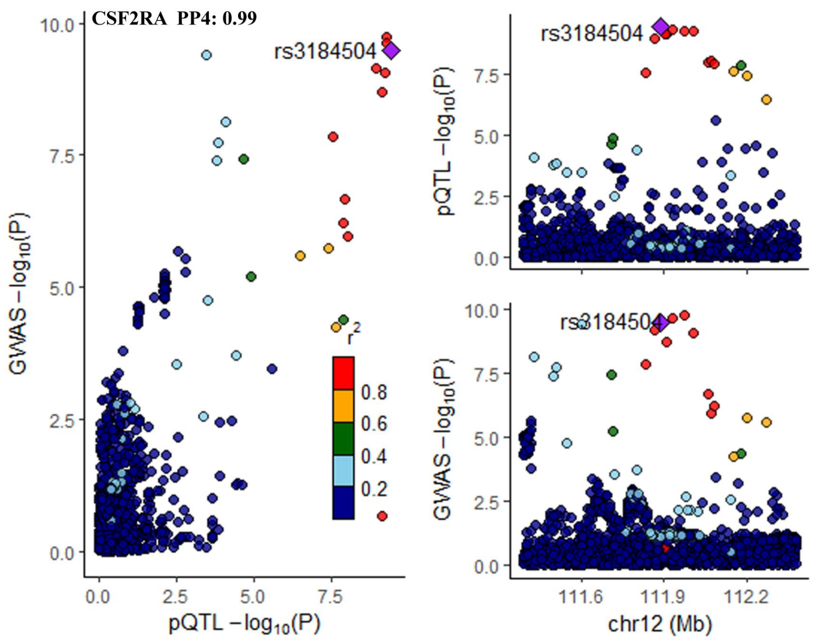

Fig S5 Regional association plot for colocalization analysis of CSF2RA protein with colorectal cancer risk. The lead SNP is shown as a purple diamond. SNPs within ±500 kb of the protein quantitative trait locus were included; p12=1e-5, prior probability a SNP is associated with both protein and CRC.

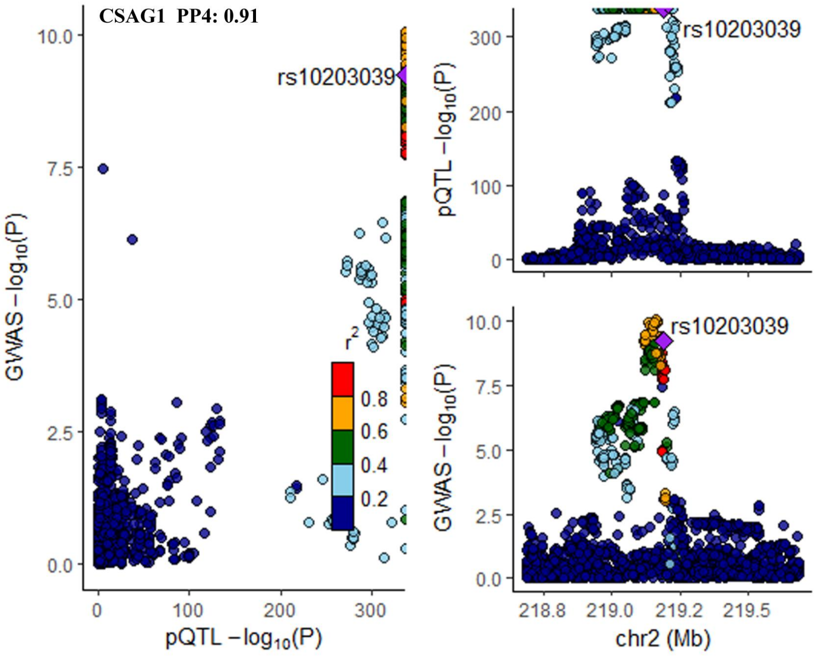

Fig S6 Regional association plot for colocalization analysis of CSAG1 protein with colorectal cancer risk. The lead SNP is shown as a purple diamond. SNPs within ±500 kb of the protein quantitative trait locus were included; p12=1e-5, prior probability a SNP is associated with both protein and CRC.

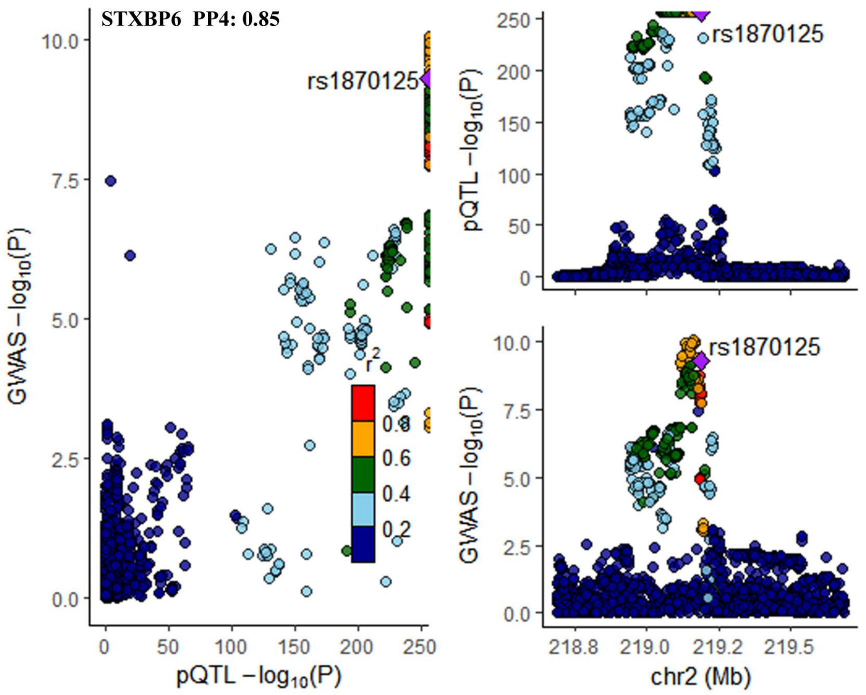

Fig S7 Regional association plot for colocalization analysis of STXBP6 protein with colorectal cancer risk. The lead SNP is shown as a purple diamond. SNPs within ±500 kb of the protein quantitative trait locus were included; p12=1e-5, prior probability a SNP is associated with both protein and CRC.

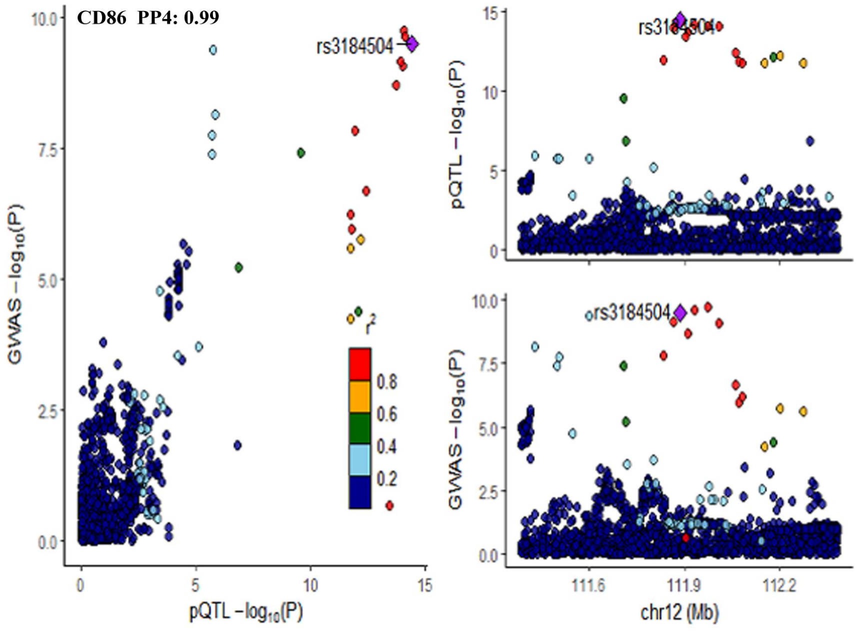

Fig S8 Regional association plot for colocalization analysis of CD86 protein with colorectal cancer risk. The lead SNP is shown as a purple diamond. SNPs within ±500 kb of the protein quantitative trait locus were included; p12=1e-5, prior probability a SNP is associated with both protein and CRC.

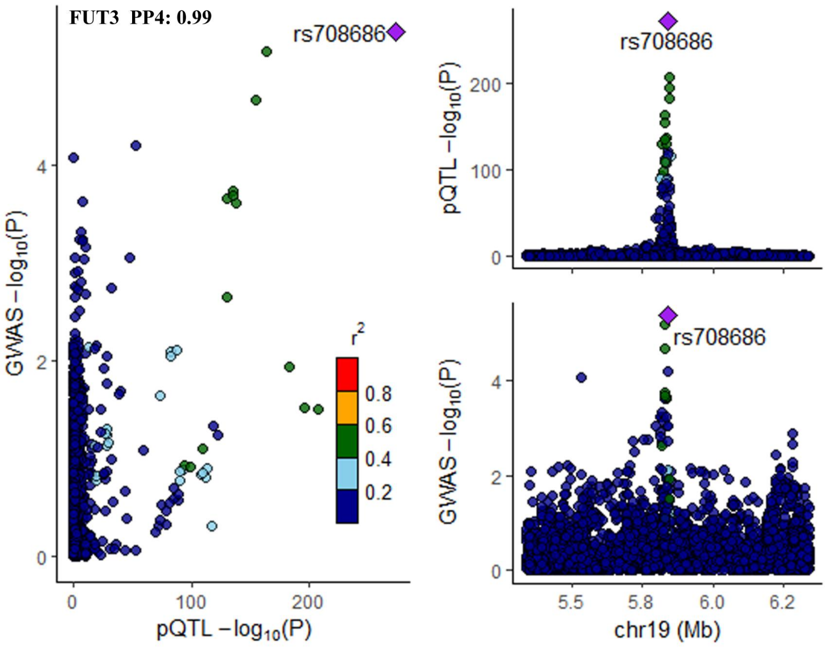

Fig S9 Regional association plot for colocalization analysis of FUT3 protein with colorectal cancer risk. The lead SNP is shown as a purple diamond. SNPs within ±500 kb of the protein quantitative trait locus were included; p12=1e-5, prior probability a SNP is associated with both protein and CRC.

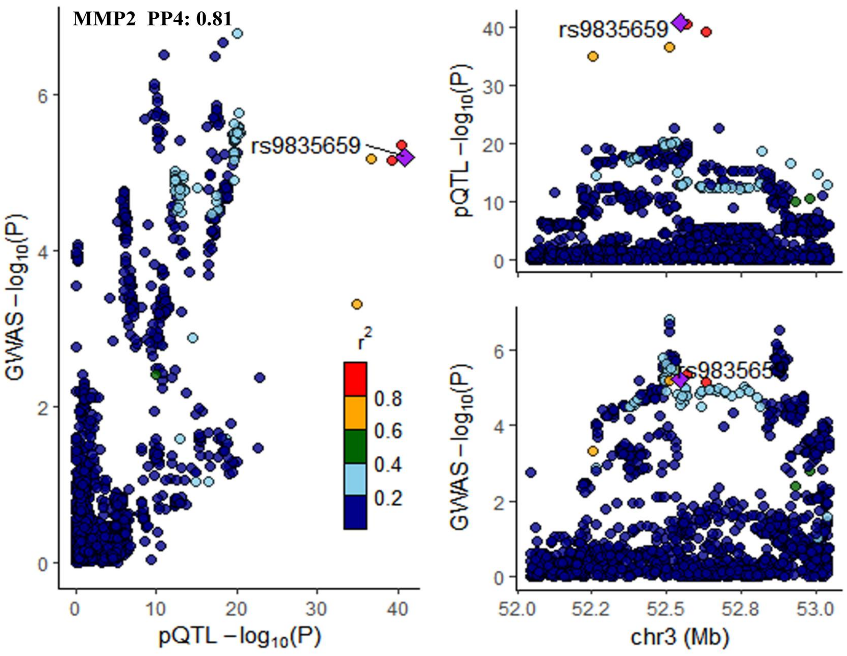

Fig S10 Regional association plot for colocalization analysis of MMP2 protein with colorectal cancer risk. The lead SNP is shown as a purple diamond. SNPs within ±500 kb of the protein quantitative trait locus were included; p12=1e-5, prior probability a SNP is associated with both protein and CRC.

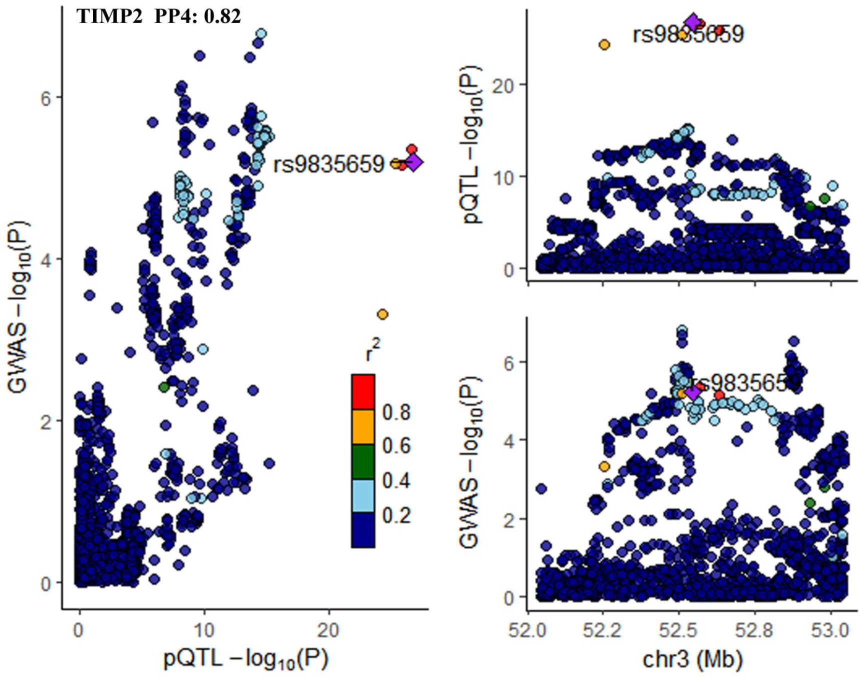

Fig S11 Regional association plot for colocalization analysis of TIMP2 protein with colorectal cancer risk. The lead SNP is shown as a purple diamond. SNPs within ±500 kb of the protein quantitative trait locus were included; p12=1e-5, prior probability a SNP is associated with both protein and CRC.

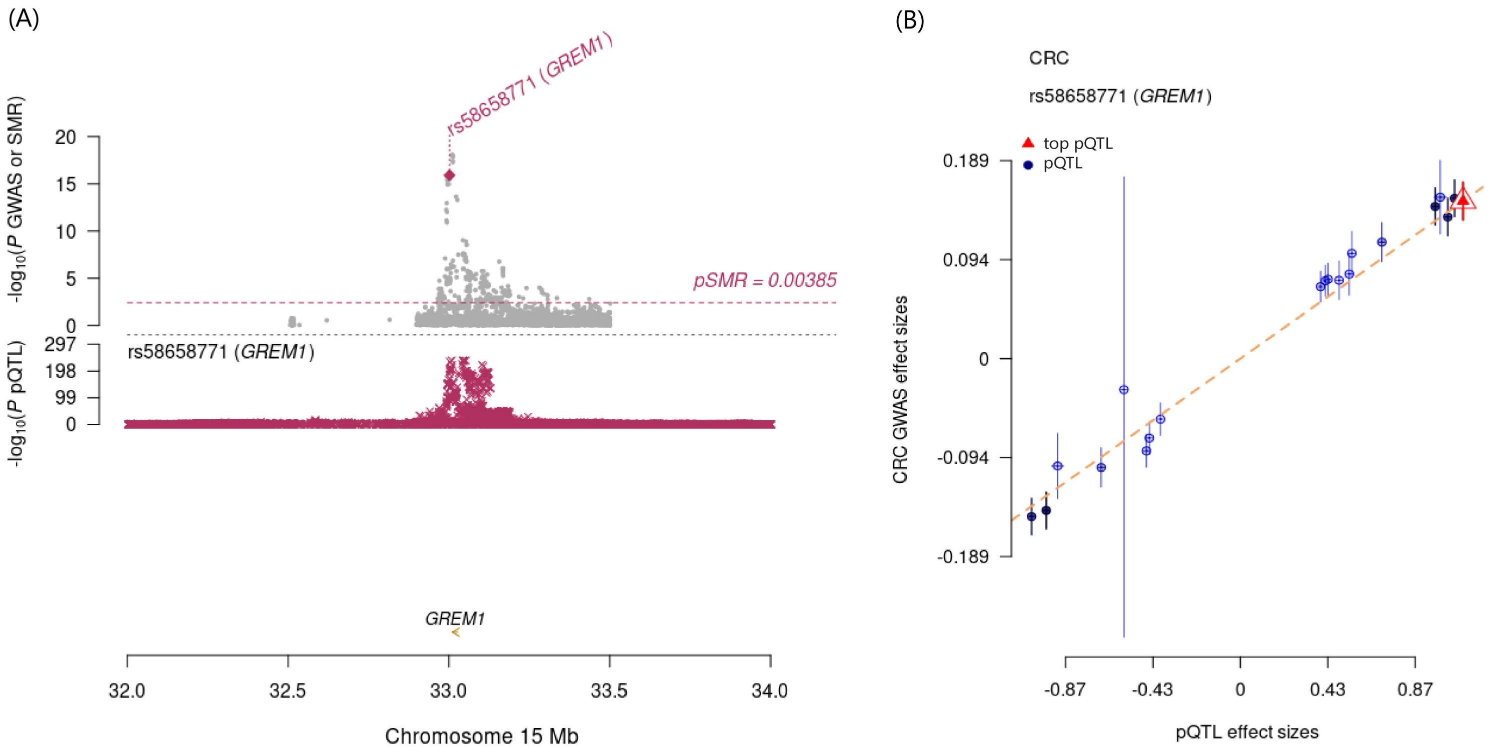

Fig S12 The summary-data-based Mendelian randomization (SMR) result of GREM1 with colorectal cancer (CRC) risk. (A) The SMR locus plot. Top plot, gray dots represent the *P* values for SNPs from the CRC meta-GWASs, diamond represents the *P* value for GREM1 from the SMR test. Bottom plot shows the summary statistics of GREM1 protein. (B) The SMR effect plot. The horizontal axis represents the effect sizes of SNPs on GREM1, while the vertical axis represents the effect sizes of SNPs on CRC risk. pOTL, protein quantitative trait locus.

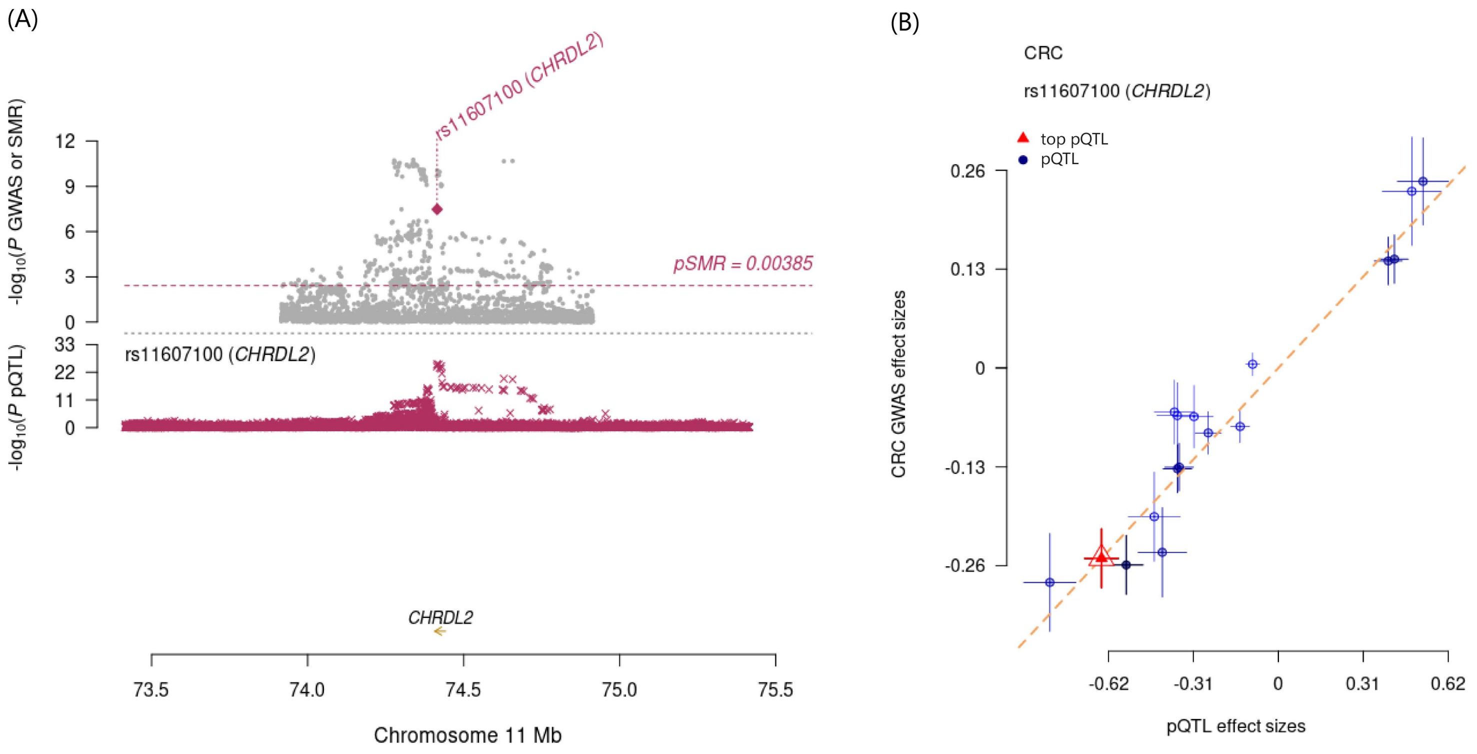

Fig S13 The summary-data-based Mendelian randomization (SMR) result of CHRDL2 with colorectal cancer (CRC) risk. (A) The SMR locus plot. Top plot, gray dots represent the *P* values for SNPs from the CRC meta-GWASs, diamond represents the *P* value for CHRDL2 from the SMR test. Bottom plot shows the summary statistics of CHRDL2 protein. (B) The SMR effect plot. The horizontal axis represents the effect sizes of SNPs on CHRDL2, while the vertical axis represents the effect sizes of SNPs on CRC risk. pOTL, protein quantitative trait locus.

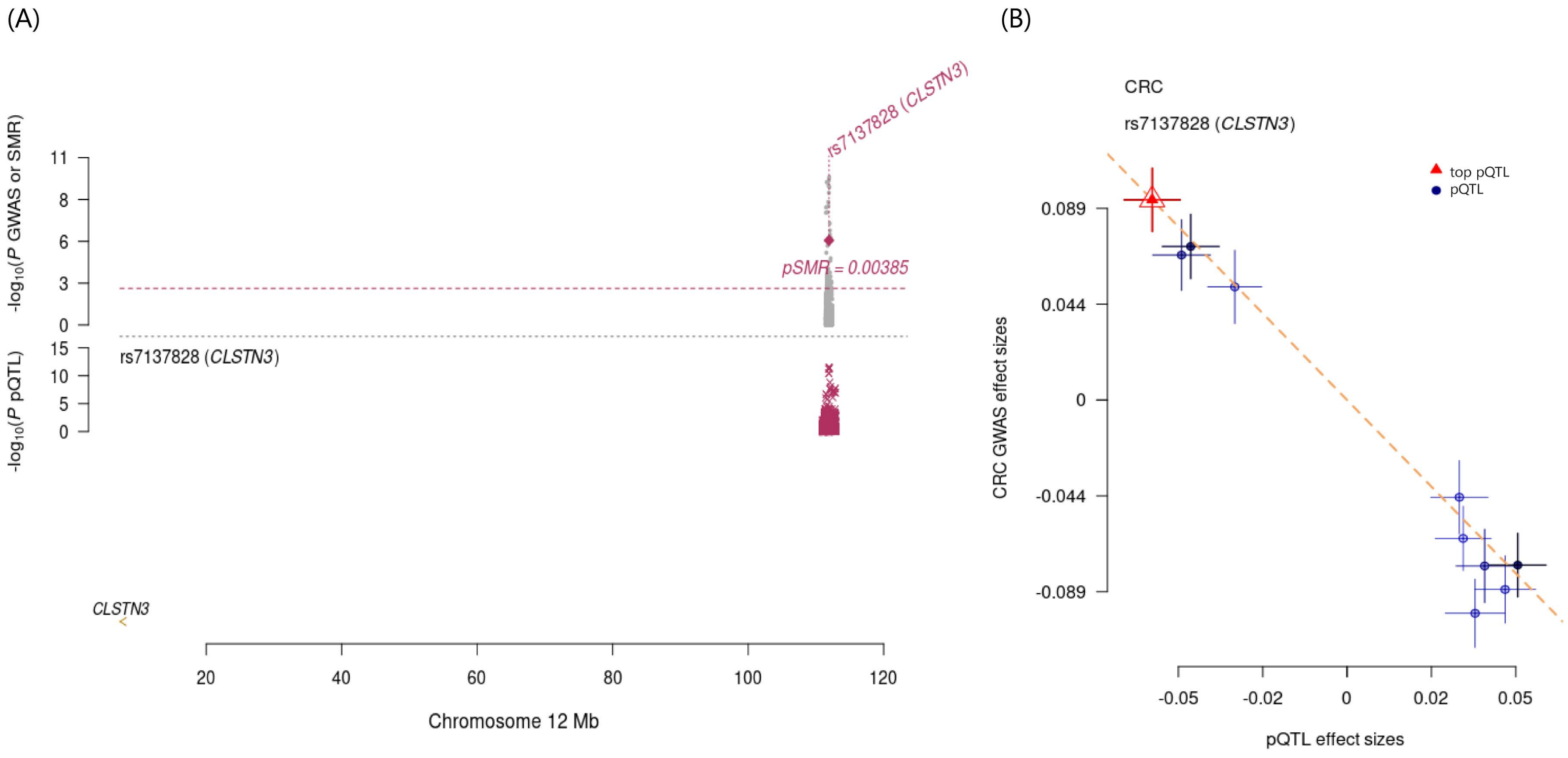

Fig S14 The summary-data-based Mendelian randomization (SMR) result of CLSTN3 with colorectal cancer (CRC) risk. (A) The SMR locus plot. Top plot, gray dots represent the *P* values for SNPs from the CRC meta-GWASs, diamond represents the *P* value for CLSTN3 from the SMR test. Bottom plot shows the summary statistics of CLSTN3 protein. (B) The SMR effect plot. The horizontal axis represents the effect sizes of SNPs on CLSTN3, while the vertical axis represents the effect sizes of SNPs on CRC risk. pOTL, protein quantitative trait locus.

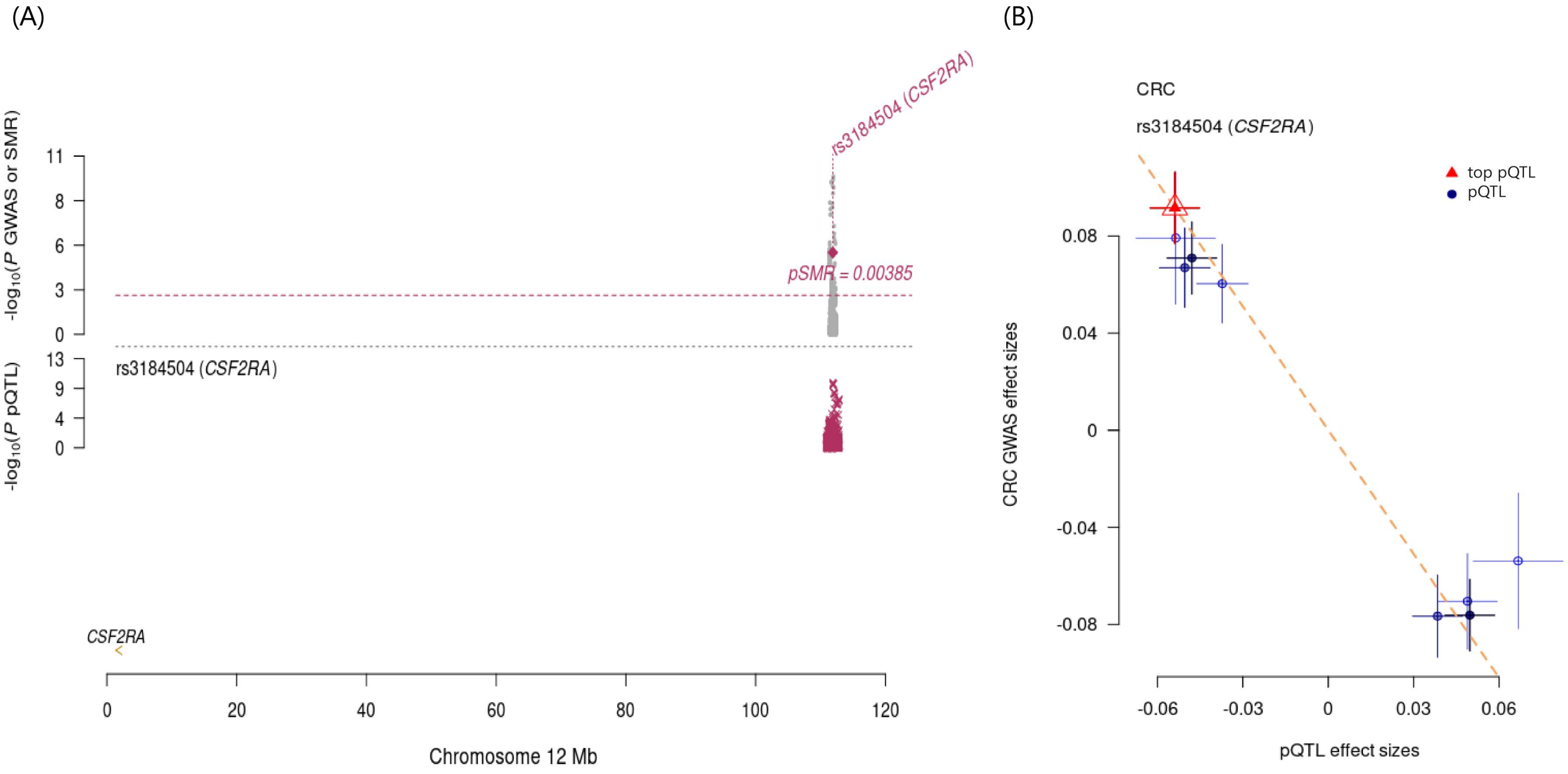

Fig S15 The summary-data-based Mendelian randomization (SMR) result of CSF2RA with colorectal cancer (CRC) risk. (A) The SMR locus plot. Top plot, gray dots represent the *P* values for SNPs from the CRC meta-GWASs, diamond represents the *P* value for CSF2RA from the SMR test. Bottom plot shows the summary statistics of CSF2RA protein. (B) The SMR effect plot. The horizontal axis represents the effect sizes of SNPs on CSF2RA, while the vertical axis represents the effect sizes of SNPs on CRC risk. pOTL, protein quantitative trait locus.

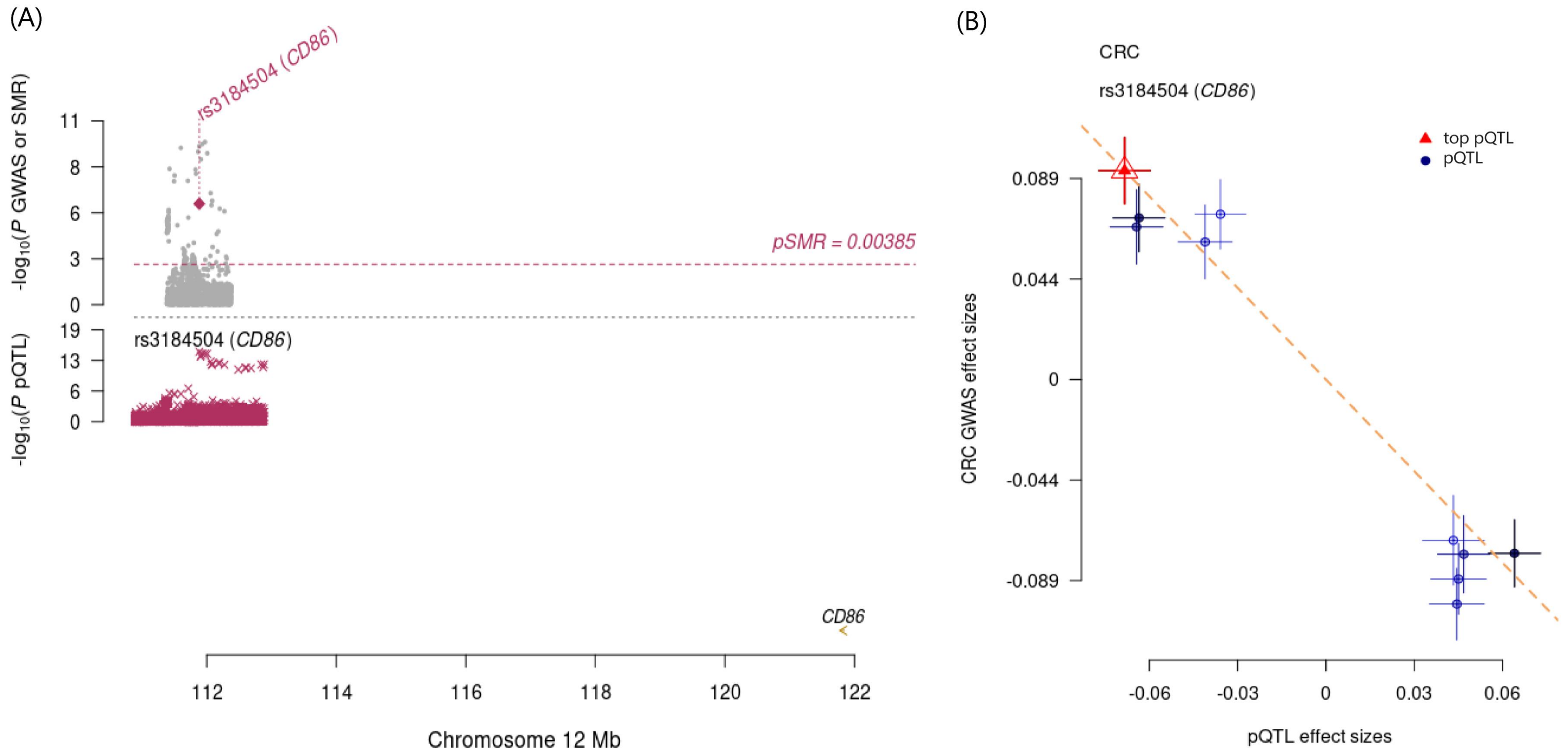

Fig S16 The summary-data-based Mendelian randomization (SMR) result of CD86 with colorectal cancer (CRC) risk. (A) The SMR locus plot. Top plot, gray dots represent the *P* values for SNPs from the CRC meta-GWASs, diamond represents the *P* value for CD86 from the SMR test. Bottom plot shows the summary statistics of CD86 protein. (B) The SMR effect plot. The horizontal axis represents the effect sizes of SNPs on CD86, while the vertical axis represents the effect sizes of SNPs on CRC risk. pOTL, protein quantitative trait locus.

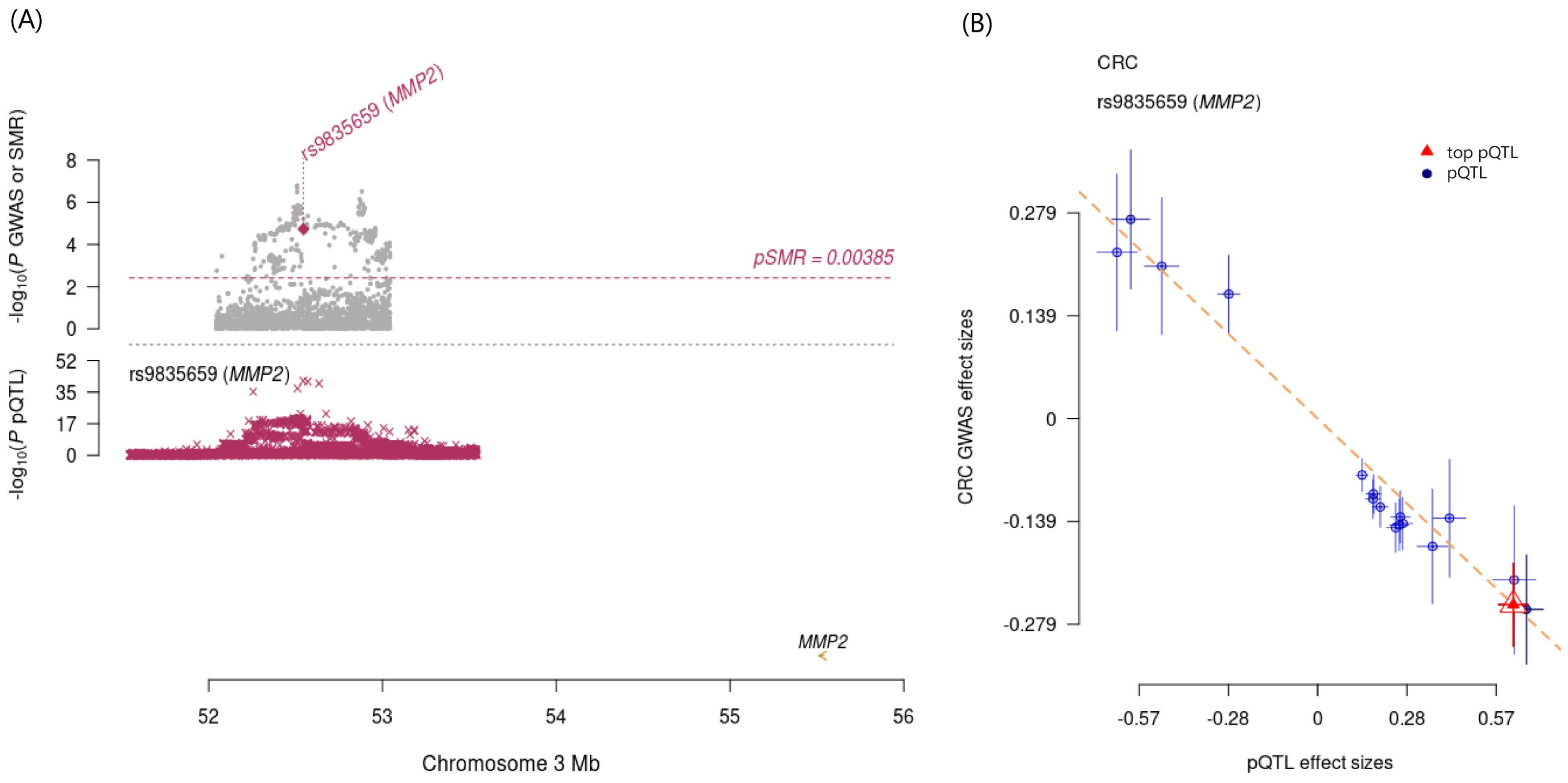

Fig S17 The summary-data-based Mendelian randomization (SMR) result of MMP2 with colorectal cancer (CRC) risk. (A) The SMR locus plot. Top plot, gray dots represent the *P* values for SNPs from the CRC meta-GWASs, diamond represents the *P* value for MMP2 from the SMR test. Bottom plot shows the summary statistics of MMP2 protein. (B) The SMR effect plot. The horizontal axis represents the effect sizes of SNPs on MMP2, while the vertical axis represents the effect sizes of SNPs on CRC risk. pOTL, protein quantitative trait locus.

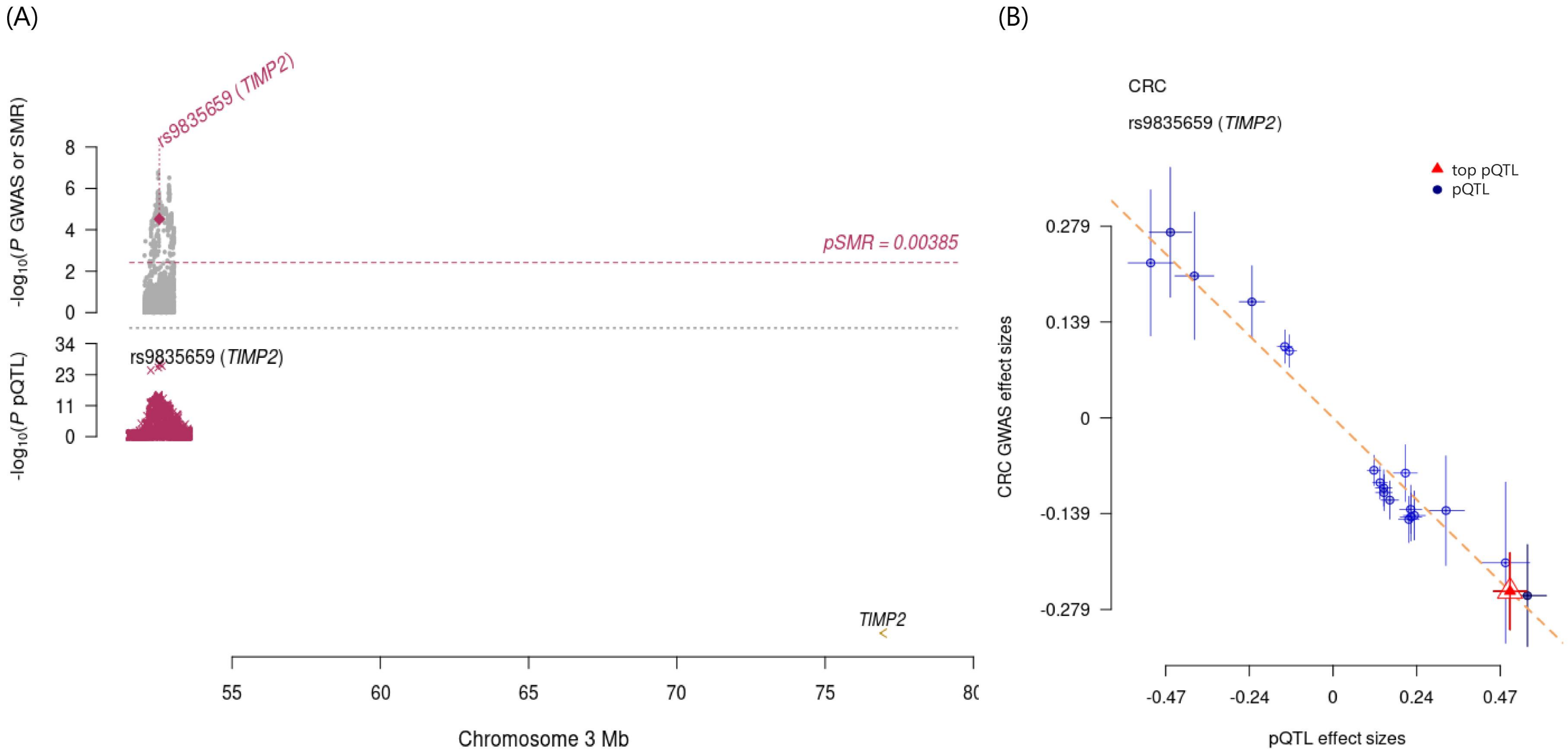

Fig S18 The summary-data-based Mendelian randomization (SMR) result of TIMP2 with colorectal cancer (CRC) risk. (A) The SMR locus plot. Top plot, gray dots represent the *P* values for SNPs from the CRC meta-GWASs, diamond represents the *P* value for TIMP2 from the SMR test. Bottom plot shows the summary statistics of TIMP2 protein. (B) The SMR effect plot. The horizontal axis represents the effect sizes of SNPs on TIMP2, while the vertical axis represents the effect sizes of SNPs on CRC risk. pOTL, protein quantitative trait locus.

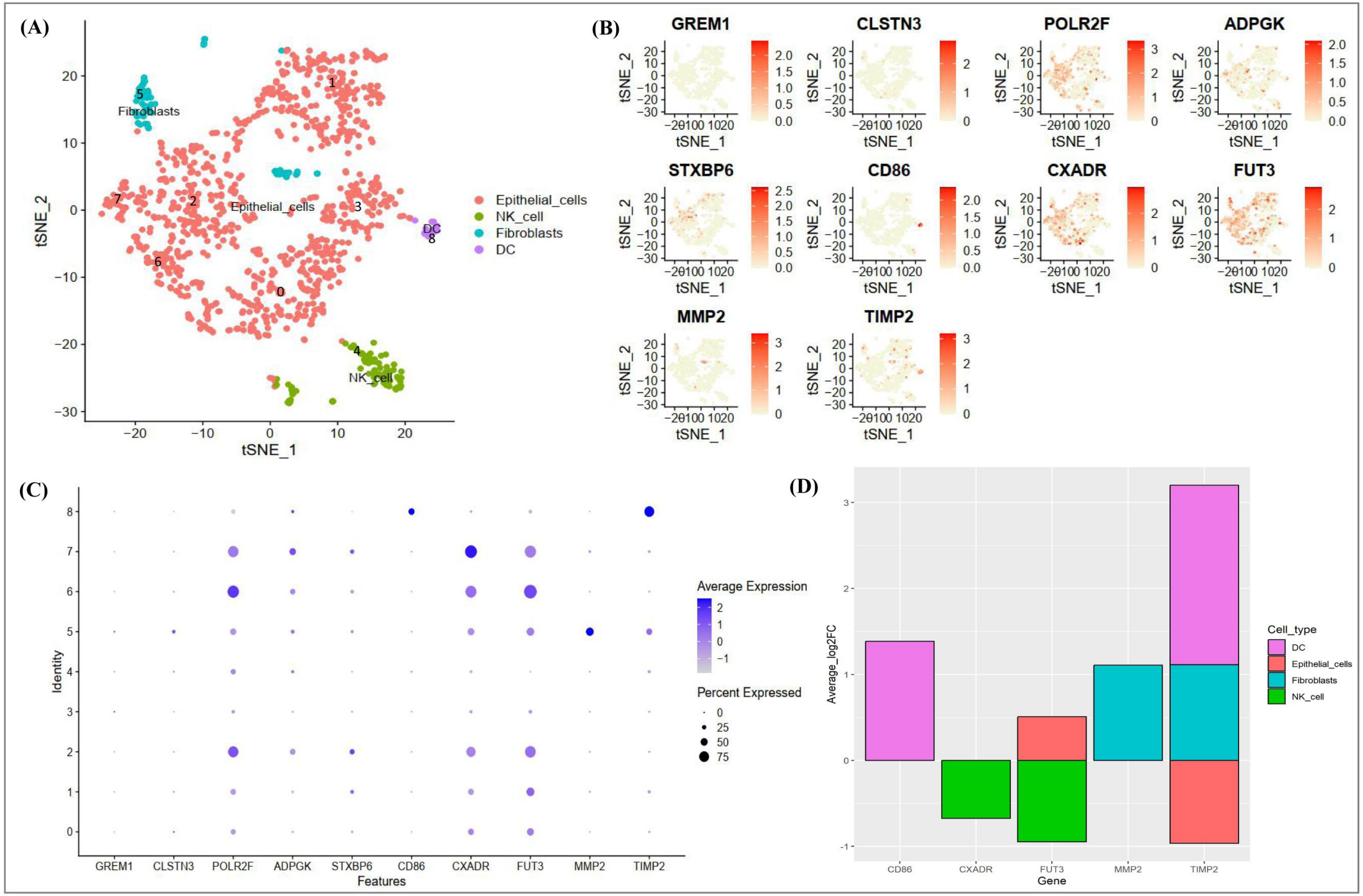

Fig S19 Single-cell type expression in normal colon tissue for the coding genes of proteins identified by proteome-wide Mendelian randomization. (A) A total of nine cell clusters and four cell types were identified. (B) and (C) show the expression of protein coding genes in each cluster. (D) Five protein-coding genes had evidence of enrichment in a cell type at average $Log_2FC > 0.5$ and $FDR < 0.05$ level. DC, dendritic cell.

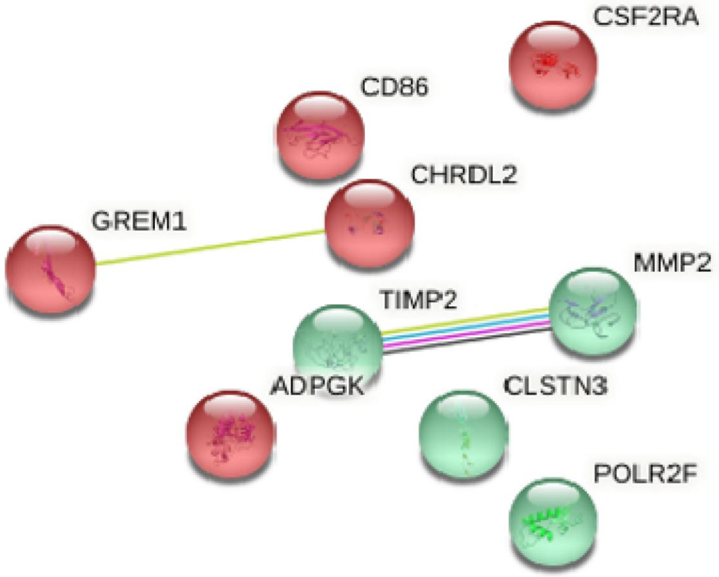

Fig S20 The Protein-protein interaction (PPI) network of proteins identified by proteome-wide Mendelian randomization. Lines represent interactions between proteins. Green line indicates gene neighborhood and predicted interaction; Blue line indicates known interaction from curated databases; Fuchsia line indicates known interaction that is experimentally determined. Black line indicates co-expression. Data information was from STRING database.
